# Proteomics reveals spatial and molecular heterogeneities in advanced atherosclerotic carotid artery plaques

**DOI:** 10.1038/s44161-026-00827-1

**Published:** 2026-06-22

**Authors:** Ankit Sinha, Nadja Sachs, Elena Kratz, Jessica Pauli, Sophia Steigerwald, Vincent Albrecht, Thierry M. Nordmann, Enes Ugur, Edwin H. Rodriguez, Marie-Luise Engl, Patricia Skowronek, Denys Oliinyk, Andreas Metousis, Moritz von Scheidt, Michael Wierer, Hanna Winter, Heribert Schunkert, Daniela Branzan, Lars Maegdefessel, Matthias Mann

**Affiliations:** 1 Max Planck Institute of Biochemistry, Martinsried, Germany; 2 German Centre for Cardiovascular Research (DZHK) Partner site Munich Heart Alliance, Munich, Germany; 3 Department for Vascular and Endovascular Surgery, TUM Klinikum, Technical University Munich, Munich, Germany; 4 Institute of Molecular Vascular Medicine, TUM Klinikum, TUM, Munich, Germany; 5 Molecular and Spatial Biology of Skin, Max Planck Institute of Biochemistry, Martinsried, Germany; 6 Department of Dermatology and Allergy, University Hospital, LMU Munich, Munich, Germany; 7 Department of Dermatology, University Hospital Zurich, Zurich, Switzerland; 8 Department of Cardiology, Deutsches Herzzentrum, TUM Klinikum, Munich, Germany; 9 Proteomics Research Infrastructure, University of Copenhagen, Copenhagen, Denmark; 10 Department of Medicine, Centre for Molecular Medicine, Karolinska Institutet, Stockholm, Sweden

**Keywords:** Proteomics, Atherosclerosis, Data integration

## Abstract

Atherosclerotic plaque rupture is a major cause of cerebrovascular events, yet the molecular determinants underlying vulnerability-related plaque morphology, including fibrous-cap thickness, remain incompletely defined. Using histomorphology-guided spatial proteomics, here we delineate molecular programs associated with plaque cap phenotype across discrete plaque subregions. In 112 carotid endarterectomy specimens, differences between thin-cap and thick-cap plaques were predominantly localized to the necrotic core and fibrous cap. These differences were enriched for processes related to inflammation, lipid handling, extracellular matrix remodeling and ossification/calcification, and supported the presence of proteome-based plaque subtypes. PCSK9 was among the proteins most strongly associated with thin-cap plaques. Consistently, an in vitro model of necrotic core-like oxidative and inflammatory stress increased PCSK9 secretion in primary vascular smooth muscle cells. Together, these findings localize molecular programs associated with cap phenotype to plaque compartments and provide a framework for spatially informed biomarker discovery in advanced carotid atherosclerosis.

## Abstract (web summary)

Atherosclerotic plaque rupture underlies many cerebrovascular events, yet the molecular determinants of vulnerable plaque morphology remain poorly defined. Using histomorphology-guided spatial proteomics of 112 carotid plaques, Sinha et al. show that cap thickness-associated molecular programs are spatially confined to the fibrous cap and necrotic core, link thin-cap plaques to inflammatory and lipid handling pathways, and identify PCSK9 as a compartment-specific marker induced by oxidative and inflammatory stress in vascular smooth muscle cells.

## Main

Carotid plaque rupture is a major cause of cerebrovascular events, yet identifying lesions with adverse morphological features remains challenging. Although severe carotid stenosis has long guided risk assessment and intervention, many acute events arise from plaques with only moderate luminal narrowing^1–3^. Attention has therefore shifted from luminal obstruction to plaque composition and morphology, as features such as intraplaque hemorrhage, large necrotic cores and reduced fibrous-cap thickness are associated with plaques prone to rupture^4^. Understanding the molecular basis of these features may improve mechanistic insight and plaque risk stratification^5^.

Minimum cap thickness (MCT) is an important morphological feature of advanced carotid plaques because the cap is the principal barrier between the thrombogenic necrotic core and circulating blood^6^. Its integrity depends on the balance between extracellular matrix (ECM) synthesis by vascular smooth muscle cells (VSMCs) and matrix degradation driven by inflammatory cells and proteolytic enzymes. In carotid plaques, reduced MCT has been associated with rupture-prone morphology, consistent with loss of structural containment and increased inflammatory injury^7–9^.

Carotid plaques are histomorphologically heterogeneous and comprise three major subregions: the tunica media, a VSMC-rich layer that provides tensile support; the necrotic core, a lipid-rich compartment containing apoptotic cells and cellular debris; and the fibrous cap, a collagen-rich barrier separating the thrombogenic core from the arterial lumen^10–12^. The fibrous cap is reinforced by fibrillar collagens produced by synthetic VSMCs, whereas the necrotic core contains inflammatory mediators and infiltrating immune cells that promote matrix degradation and local injury^13,14^. By contrast, the media is enriched in contractile VSMCs and organized ECM that maintain arterial wall structure^15^. Molecular characterization of these subregions is therefore important for defining biological processes associated with reduced fibrous-cap thickness.

Recent bulk transcriptomic and proteomic studies have identified molecular phenotypes of carotid atherosclerotic plaques characterized by inflammation, lipid metabolism, ECM remodeling and calcification^16–18^. However, the mapping of molecular patterns onto histomorphological subregions and in relation to cap thickness remains unclear^19^. To address this gap, we applied mass spectrometry-based spatial proteomics to profile protein changes across the three major subregions of advanced carotid plaques stratified by MCT into thin-cap (TnC; <200 µm) and thick-cap (TkC; ≥200 µm) plaques^6,20^. This approach links molecular programs to plaque microanatomy and enables prioritization of candidate markers associated with the TnC plaque phenotype.

## Results

### Study design, clinical cohorts and data preparation

**Fig. 1:**
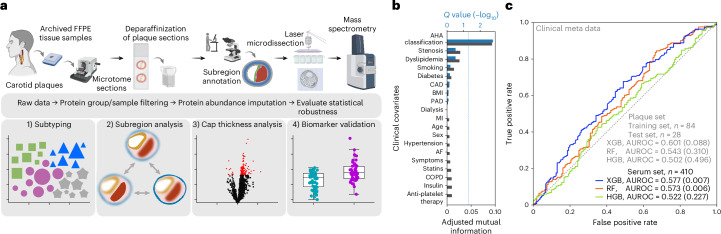
Clinical metadata show limited discrimination of carotid plaque MCT status. **a**, Histomorphology-guided spatial proteomics workflow for CEA plaques (*n* = 112). Formalin-fixed paraffin-embedded (FFPE) plaques were sectioned, deparaffinized, histologically annotated, microdissected by subregion (media, fibrous cap and necrotic core) and analyzed by liquid chromatography coupled to tandem mass spectrometry (LC–MS/MS), followed by quality control, imputation and downstream analyses. Areas with minimal intraplaque hemorrhage were preferentially sampled. **b**, Clinical covariates ranked by association with MCT status using adjusted mutual information (AMI). Blue bars indicate significance after multiple-testing correction; dotted line indicates *q* = 0.05. AF, atrial fibrillation; AHA, American Heart Association; BMI, body mass index; CAD, coronary artery disease; COPD, chronic obstructive pulmonary disease; MI, myocardial infarction; PAD, peripheral artery disease. **c**, AUROC performance of clinical metadata for discriminating MCT status using XGBoost (XGB), random forest (RF) and gradient boosting (HistGradientBoost or HGB) in the plaque cohort (training, *n* = 84; test, *n* = 28) and serum cohort (*n* = 410). Illustration in **a** created in BioRender. Sinha, A; https://biorender.com/9c0tapz (2026). Source data

To identify protein signatures associated with MCT status in advanced carotid plaques, we established a histomorphology-guided proteomics workflow (Fig. 1a). Under pathologist guidance, we used laser microdissection to isolate spatially distinct plaque subregions (the media, necrotic core and fibrous cap) from carotid plaques obtained from patients undergoing carotid endarterectomy (CEA). We then profiled the proteome of each subregion from each plaque using a customized ion mobility mass spectrometry acquisition method^21^. This workflow enabled spatially resolved sampling while preserving the anatomical context of protein abundance patterns in TkC and TnC plaques.

We applied this workflow to carotid plaques from 112 patients. Cases were selected on the basis of completeness of clinical data, balance of clinical covariates across MCT status and histological integrity of the media subregion. Clinical covariates were matched to improve comparability between the primary endpoint groups: TkC plaques (*n* = 55, cap thickness ≥ 200 μm) and TnC plaques (*n* = 57, cap thickness < 200 μm)^6,20^. Secondary covariates included dyslipidemia, diabetes, body mass index (BMI), sex, symptom status (asymptomatic versus symptomatic, defined by transient ischemic attack, amaurosis fugax or stroke), medication use and lifestyle history (Supplementary Table 1). Overall, 94% of cases had an American Heart Association (AHA) histological classification of V or higher, and 79% had severe stenosis (≥80% luminal occlusion)^22^.

Among the clinical covariates assessed, AHA classification was the only variable associated with MCT status, and the association was modest (adjusted mutual information (AMI) = 0.095, *P* = 0.003, permutation test; Fig. 1b). By contrast, stenosis severity was similarly distributed between TnC and TkC plaques (*P* > 0.15), reducing the likelihood that subsequent analyses were driven primarily by differences in luminal narrowing. Clinical covariates, whether considered individually or in combination, showed limited discrimination of MCT status (area under the receiver operating characteristic curve or AUROC = 0.50–0.60; *n* = 28 models; Fig. 1c). Similar performance was observed in a larger serum-only cohort (AUROC = 0.52–0.58; *n* = 410; Fig. 1c and Supplementary Table 1). Together, these findings indicate that clinical metadata alone provide limited discrimination of plaque MCT status and support the search for protein-based signatures.

### Quantitative patterns in plaque proteomes

**Fig. 2:**
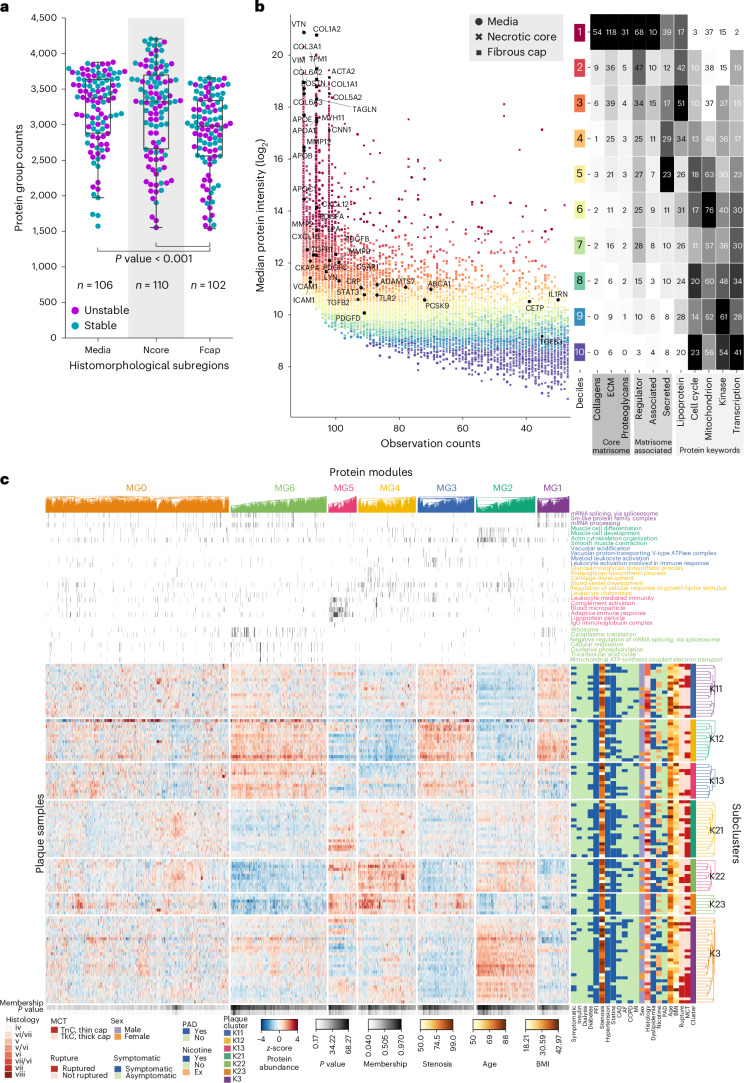
Plaque subregion proteomes show distinct abundance structure and necrotic core subtypes. **a**, Numbers of quantified proteins per sample across plaque subregions after quality control. Proteins with >66% missing values and samples with <1,500 quantified proteins were excluded. Sample sizes are indicated below each subregion. Points indicate individual plaque samples, boxes indicate the median and interquartile range, and whiskers show the data range, except for outlier points, which were determined using the interquartile range. Significance (*P* < 0.0001) was determined using a *t*-test. Fcap, fibrous cap; Ncore, necrotic core. **b**, Protein detection and abundance characteristics across plaque subregions. The scatterplot shows median protein intensity (log_2_) versus observation count, with points colored by abundance decile; selected proteins are annotated. The accompanying heat map summarizes the distribution of functional protein classes across abundance deciles. **c**, Consensus clustering of necrotic core proteomes (*n* = 110) using the top 66% most variable proteins identified seven subclusters (*K* = 7; Extended Data Fig. 2). The heat map shows *z*-scored protein abundances (red, higher; blue, lower), organized by weighted correlation network analysis-derived protein modules (MG0–MG6; top annotation). MG0 denotes low-membership or unassigned proteins. Bottom annotations indicate module membership strength and statistical significance. Clinical covariates and plaque characteristics are shown as row annotations (right). PAD, peripheral artery disease; PFI, platelet function inhibition. Source data

**Extended Data Fig. 1:**
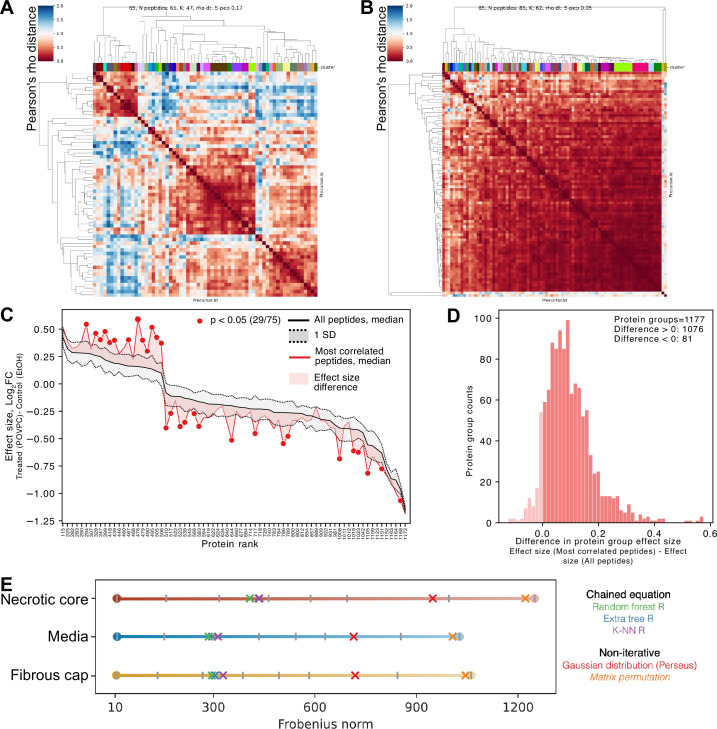
Refinement and quality assessment of quantitative proteomics measurements. **A–B**, Internal consistency analysis (ICA) illustrated by peptide–peptide correlation heatmaps for two representative proteins: A, EEF2 (low internal consistency) and B, PPL (high internal consistency). Heatmaps show pairwise peptide correlations (Pearson’s ρ) with hierarchical clustering; peptide clusters (K) were defined using a weighted Pearson-distance threshold (*dt*) as indicated in each panel. **C**, Comparison of protein effect sizes (log2 fold change, POVPC vs ethanol control) estimated using all peptides (black, median fold change) versus the most-correlated peptide subset defined by ICA (red, median fold change). Shaded bands indicate the effect-size difference between approaches; dotted lines show ±1 SD from bootstrap resampling (1,001 iterations, resampling size = number of correlated peptides). Red points mark proteins with significantly different effect-size estimates (P < 0.05; 29 of 75 proteins tested, moderated F-statistic test). **D**, Distribution of protein-level effect-size differences across protein groups, calculated as effect size (most-correlated peptides) − effect size (all peptides), demonstrating a predominant shift toward larger absolute effects when restricting to the most internally consistent peptides (positive differences: 1,076 of 1,177 proteins). **E**, Benchmarking of missing-value imputation strategies using the Frobenius norm to quantify imputation-induced matrix distortion for each plaque subregion. Chained-equation (iterative) approaches (random forest, extra trees, k-nearest neighbours) are compared with non-iterative methods (Gaussian imputation as implemented in Perseus; matrix permutation). Lower Frobenius norms indicate reduced imputation artefacts.

Our proteomic analysis identified 4,893 unique proteins across all plaque subregions. After subregion-specific filtering based on observation counts, an average of 3,412 proteins were quantified in the media (*n* = 106), 3,321 in the necrotic core (*n* = 110) and 2,957 in the fibrous cap (*n* = 102) from TkC and TnC plaques (Fig. 2a). To generate the protein quantification matrix, we modified the quantification step by calculating protein intensities using only highly correlated peptides, defined by density-weighted Pearson correlation distance. This exclusion-based approach was used to reduce the impact of sequence complexity arising from extensive proteoform heterogeneity, particularly in extracellular and cell-surface proteins and in atherosclerosis-relevant proteins such as LPA (Extended Data Fig. 1a,b)^23^. When evaluated in a cellular proteomic dataset with known treatment and control conditions, this approach increased effect sizes among significantly altered proteins (Extended Data Fig. 1c,d). To generate the final matrix, we imputed missing values using a random-forest-based chained equations method, which yielded the smallest estimated imputation artifact (Extended Data Fig. 1e and Supplementary Table 2)^24^.

To characterize the dominant protein constituents of carotid plaques and assess how these major components were represented in the mass spectrometry measurements, we stratified proteins by abundance decile, based on median mass spectrometry intensity, and by functional category using UniProt keywords. This analysis revealed marked functional stratification across the plaque proteome, with structural proteins enriched in the highest-abundance decile (decile 1; Fig. 2b). These included ECM proteins, such as VTN, COL1A2, COL3A1 and COL6A2, as well as VSMC contractile markers, including VIM, TPM1, ACTA2, TAGLN and MYH11 (Fig. 2b). Proteins involved in ECM remodeling and inflammatory or immune signaling were more prevalent in intermediate-abundance deciles (2–7), whereas intracellular regulatory proteins, including cell-cycle regulators and kinases, were more often detected in lower-abundance deciles (6–10)^25^. Overall, these patterns are consistent with established plaque biology and indicate that the proteomic measurements captured both abundant structural components and lower-abundance regulatory proteins.

**Extended Data Fig. 2:**
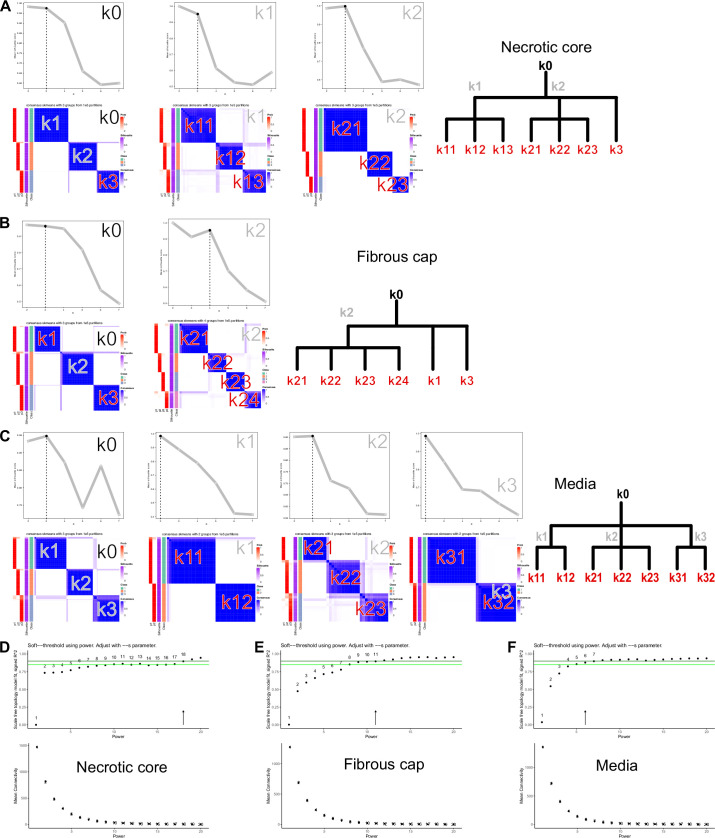
Identification of proteome-defined subclusters within plaque subregions and construction of protein co-expression modules. **A–C**, Proteome-based hierarchical subclustering of plaque subregions: **A**, necrotic core, **B**, fibrous cap, and **C**, media. For each subregion, the right-hand dendrogram summarises the hierarchical partitioning used for consensus clustering, with the root node (k0, black), internal nodes (grey), and terminal subclusters (red). Subcluster labels are hierarchical identifiers that encode parent–child relationships rather than an ordinal ranking: first-level clusters are denoted k1–k3, and second-level subtypes are denoted by appending a second digit (for example, k1 → k11/k12/k13; k2 → k21/k22/k23), indicating subtypes derived from the same parent and therefore more closely related. For each hierarchical level, the top plots show optimisation of the number of clusters (silhouette scores, dotted line indicates the selected solution based on highest score), and the bottom panels show consensus matrices (100,000 iterations) summarising co-clustering stability. **D–F**, Weighted gene correlation network analysis (WGCNA) performed separately within each subregion. Soft-thresholding power (β) was selected to approximate scale-free topology (top; linear regression, target fit R^2^ > 0.9), and the corresponding connectivity profiles at the chosen β are shown below (bottom), supporting the identification of distinct, subregion-specific protein co-expression modules.

**Extended Data Fig. 3:**
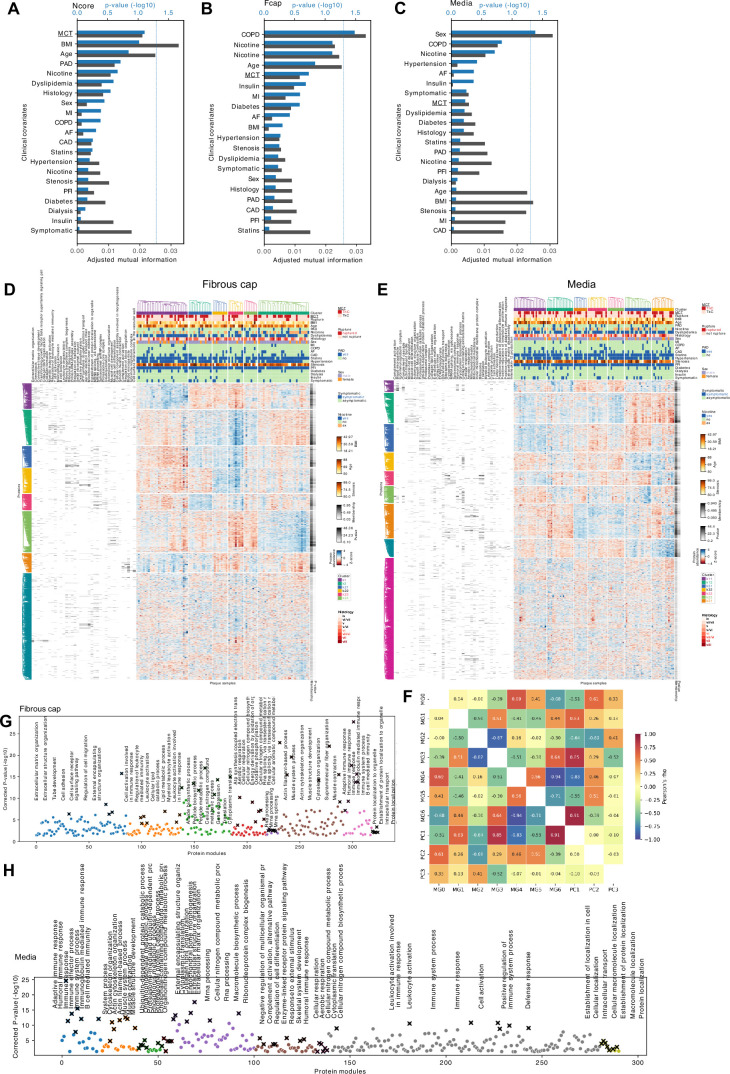
Clinical correlates of subregion subclusters and functional annotation of protein modules. **A–C**, Association of clinical covariates with proteome-defined subclusters in necrotic core (**A**), fibrous cap (**B**), and media (**C**), quantified by adjusted mutual information (black bars). Blue bars denote covariates reaching statistical significance after multiple-testing correction (q < 0.05; dotted line indicates the significance threshold on the −log10 scale). Among the tested covariates, significant associations were observed for COPD with fibrous-cap subclusters and sex with media subclusters. **D–E**, Consensus clustering of fibrous cap (**D**) and media (**E**) samples using the top 66% most variable proteins, showing subcluster structure and corresponding sample annotations. Heatmaps display z-scored protein abundances (red, higher; blue, lower) with hierarchical clustering; annotated tracks summarise clinical and histopathological metadata. **F**, Pairwise Pearson correlations between module-group summaries (MG0–MG6) and the leading PCs of the necrotic-core proteome (PC1–PC3), visualised as a correlation heatmap (values shown in cells). **G–H**, Gene Ontology enrichment of protein modules in the fibrous cap (**G**) and media (**H**), displayed as Manhattan-style plots of corrected P values (−log10). For each module, the seven most significantly enriched terms were selected for module annotation (marked by x).

Having established that the measured abundance patterns were broadly consistent with known functional programs in plaques, we next examined whether individual plaque subregions exhibited local proteomic subtypes associated with specific clinical covariates or biological processes. In necrotic core proteomes, unsupervised recursive consensus clustering identified seven subtypes (K11–K13, K21–K23 and K3; Fig. 2c and Extended Data Fig. 2a). BMI (AMI = 0.03, *P* = 0.10), age (AMI = 0.025, *P* = 0.14) and MCT status (AMI = 0.021, *P* = 0.08) showed the strongest associations with these necrotic core subtypes, but none reached statistical significance (Extended Data Fig. 3a). By contrast, fibrous-cap subtypes (*K* = 6; Extended Data Fig. 2b) were associated with chronic obstructive pulmonary disease (AMI = 0.032, *P* = 0.031; Extended Data Fig. 3b,d), whereas media subtypes (*K* = 7; Extended Data Fig. 2c) were associated with sex (AMI = 0.031, *P* = 0.040; Extended Data Fig. 3c,e). Because subtype assignments were derived independently within each subregion, different subtype combinations could occur within the same plaque. This supports the interpretation that plaque heterogeneity is spatially compartmentalized rather than uniform across the lesion. Correlation analysis of necrotic core module consensus profiles with principal components of the necrotic core proteome showed weaker correspondence for molecular group (MG) 5 and MG0 than for the other modules (Extended Data Fig. 3f).

### Functional processes define plaque subclusters

**Fig. 3:**
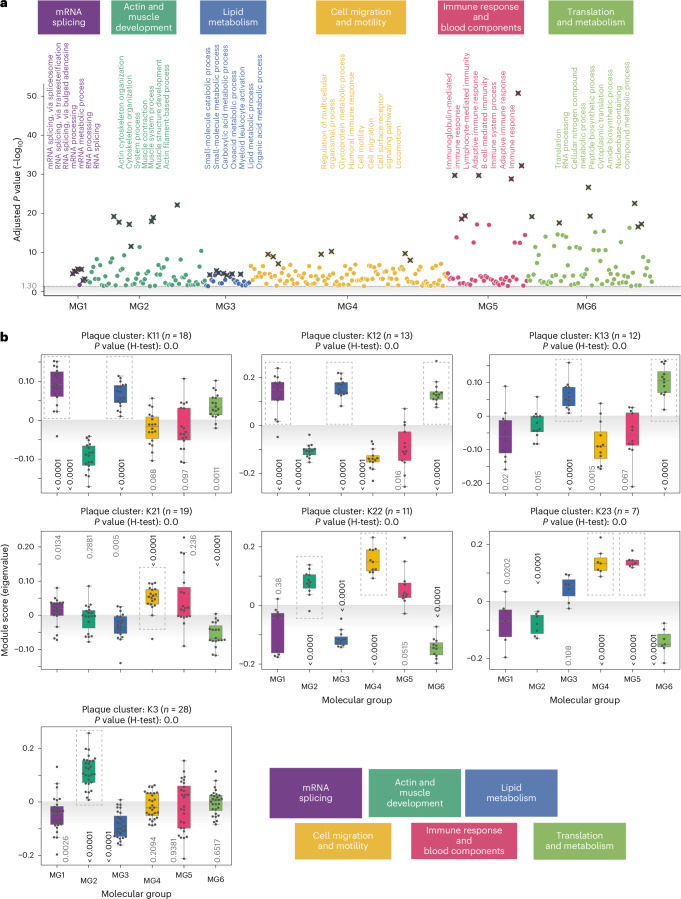
Necrotic core subclusters show distinct functional enrichments. **a**, Gene Ontology (GO) enrichment analysis of protein modules (MG1–MG6) identified in necrotic core proteomes. Points represent enriched GO terms, plotted by adjusted *P* value (Fisher’s one-sided test, −log_10_) and grouped by module; colors indicate major functional themes. **b**, Distributions of module scores across necrotic core subclusters (K11–K23 and K3). Module scores (eigengenes) summarize the correlation-weighted aggregate abundance of proteins within each MG (Kruskal–Wallis test, *P* < 1 × 10^−7^). Dashed boxes indicate modules with significantly increased scores within a given subcluster (one-sided bootstrap test, *P* < 0.0001). MG5 was enriched for blood-related and immune-related proteins and showed higher module scores in the K23 subcluster; the corresponding MG5 proteins are listed in Supplementary Table 9. Points indicate individual plaque samples, boxes indicate the median and interquartile range, and whiskers show the data range, except for outlier points, which were determined using the interquartile range. Source data

To characterize the biological programs underlying the plaque subtypes, we tested the association between the detected plaque subtypes and broader biological processes. Proteins were grouped into co-abundance modules (MGs) using weighted correlation network analysis (necrotic core: MG0–MG6; Fig. 2c, Extended Data Fig. 2d and Supplementary Table 3; fibrous cap and media: Extended Data Figs. 2e,f and 3d,e and Supplementary Tables 4 and 5). For each module, we then assessed enrichment of biological processes (Fig. 3a). In the necrotic core, MG1 was enriched for mRNA splicing and spliceosomal small nuclear ribonucleoprotein components, whereas MG2 was enriched for actin cytoskeleton and muscle contraction pathways. The remaining modules were enriched for lipid metabolism (MG3), cell migration and motility (MG4), immune response and blood-associated components (MG5) and translation/metabolism (MG6). We assigned thematic labels to each module by summarizing the seven most significantly enriched biological processes. As expected, MG0 showed no clear enrichment pattern, consistent with the absence of a coherent consensus correlation structure among its constituent proteins. Modules identified in the fibrous cap and media showed related functional themes, although their size and protein composition differed (Extended Data Fig. 3g,h).

**Extended Data Fig. 4:**
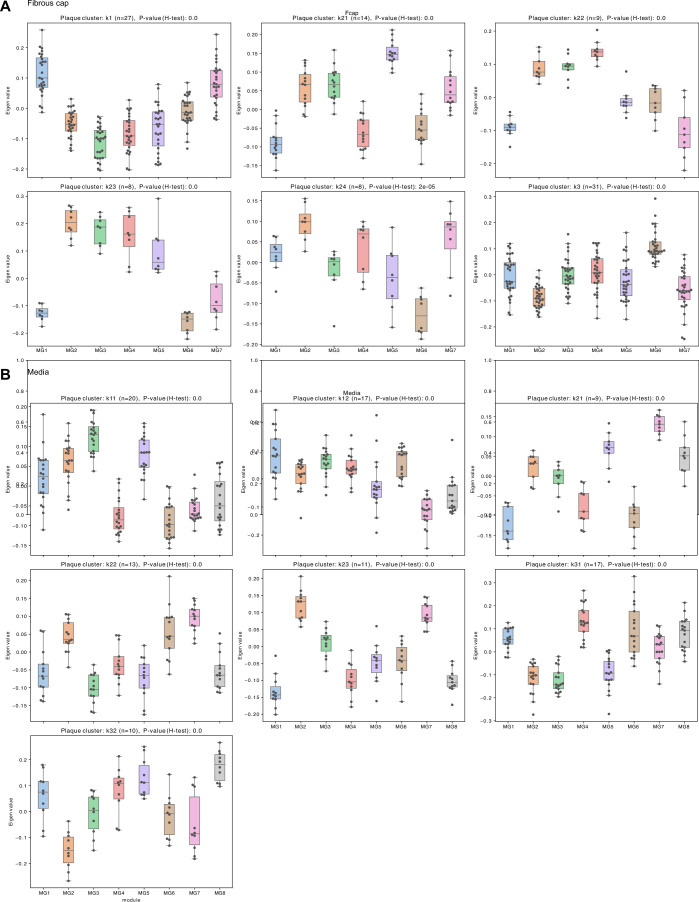
Molecular-group programmes differ across subclusters in the fibrous cap and media. **A**, Box plots showing molecular-group scores (module eigengenes) for the WGCNA-derived module groups across proteome-defined fibrous-cap subclusters (k1, k21–k24, and k3). Each panel corresponds to one subcluster and reports the sample size (n) and overall significance of between-module variation (Kruskal–Wallis H-test P value). Points represent individual samples; boxes indicate median and interquartile range, with whiskers showing the data range. **B**, Corresponding analysis in the media, showing molecular-group eigengene distributions across media subclusters (k11, k12, k21–k23, k31, and k32) with sample sizes and Kruskal–Wallis H-test P values displayed for each subcluster. Points represent individual samples; boxes indicate median and interquartile range, with whiskers showing the data range.

To interpret the plaque subtypes in biological terms, we next summarized the activity of each module using module scores (Fig. 3b). All seven necrotic core subtypes showed distinct module score profiles (*P* < 1 × 10^−7^, Kruskal–Wallis test). Within each subtype, modules with significantly elevated scores relative to a null distribution (one-sided bootstrap *P* < 0.0001) were considered dominant biological programs. Similar analyses in the fibrous cap and media also identified subregion-specific module patterns (Extended Data Fig. 4a,b)^26^. Together, these findings support the view that biological programs in advanced plaques are not uniformly distributed across plaque compartments.

### Molecular and functional characteristics of plaque subregions

**Fig. 4:**
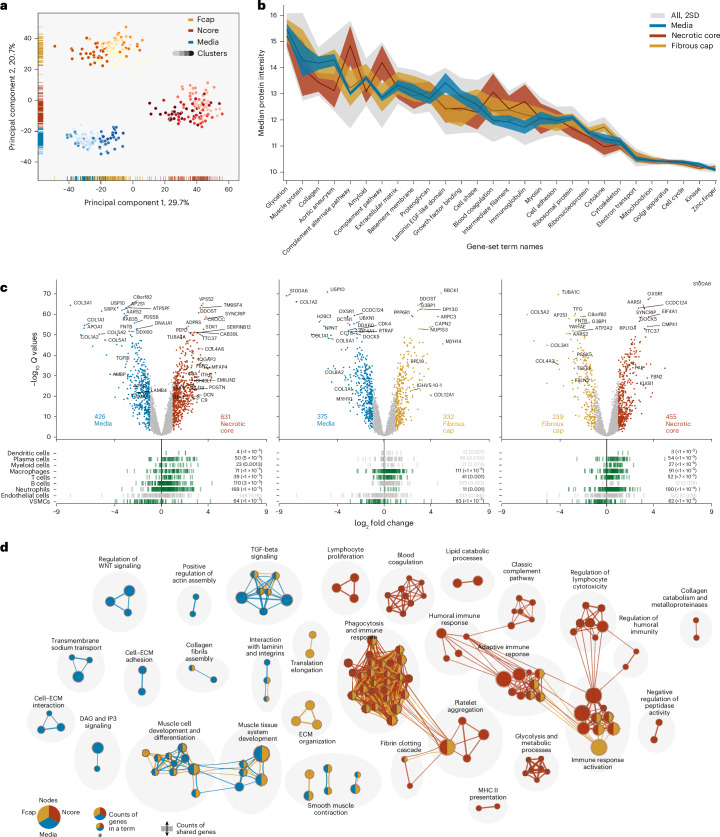
Plaque subregions show distinct proteomic and inferred cellular programs. **a**, Principal component analysis of plaque proteomes (*n* = 318 samples) showing separation by histomorphological subregion (media, necrotic core and fibrous cap); shading indicates subregion-specific clusters. **b**, Subregion-specific protein abundance patterns across functional gene-set categories. Solid lines indicate median protein intensity, and shaded ribbons denote ±2 s.d.; the gray band shows the overall ±2 s.d. across all samples. **c**, Differential protein abundance between paired subregions (*q* < 0.05, |log_2_ fold change | >1) identifies distinct molecular signatures. Lower panels show inferred cell-type enrichment based on marker proteins; significance was assessed by bootstrap testing. **d**, Network representation of enriched biological processes across plaque subregions. Nodes represent enriched terms, with pie charts indicating subregional contribution, and edges connect related pathways on the basis of shared proteins. MHC, major histocompatibility complex. Source data

Given the distinct histomorphological architecture of the three plaque subregions, we next examined the relationship between protein abundance patterns and subregion identity. Variance-based analysis showed that the necrotic core was the most molecularly distinct subregion (Fig. 4a). The first principal component (PC1; 29.7% of total variance) primarily separated the necrotic core (PC1 range: 20–60), whereas the second principal component (PC2; 20.7% of total variance) distinguished the media (PC2 range: −20 to −40) from the fibrous cap (PC2 range: 20–40).

To characterize the biological features associated with each subregion, we tested the relationship between protein abundance and functional categories (Fig. 4b)^27^. In the media, highly abundant proteins were enriched for muscle contraction, collagen and proteoglycan-related categories (*q* < 0.01), consistent with its structural role and enrichment for VSMC contractile proteins. By contrast, immune and inflammatory categories predominated in the necrotic core (*q* < 0.01). The fibrous cap showed an intermediate profile, combining features of the media and necrotic core, including cell–ECM interaction and inflammatory categories (*q* < 0.01). Intracellular signaling and regulatory proteins, including kinases and cell-cycle-associated factors, were generally detected at lower abundance and with lower variance across subregions.

Pairwise comparisons further demonstrated subregion-specific protein abundance patterns (Fig. 4c). The necrotic core showed the most distinct profile, with 631 and 455 proteins increased relative to the media and fibrous cap, respectively (*q* < 0.05). These included inflammatory and immune-associated proteins, such as CD1, ITIH2 and C9, as well as ECM-remodeling proteins, including POSTN. Fewer proteins were selectively increased in the media (375) or fibrous cap (332). Proteins enriched in the media, including COL3A1, COL1A2 and COL5A2, were consistent with its contribution to arterial structure and VSMC-rich composition, whereas proteins enriched in the fibrous cap, including COL12A1 and RPL19, were consistent with its distinct structural and cellular profile.

Inferred cell-type associations were also consistent with these subregional differences (Fig. 4c)^28,29^. The necrotic core was enriched for macrophage, neutrophil and other immune cell markers (*P* < 0.002), in keeping with its inflammatory profile. By contrast, the media was enriched for VSMC markers (*P* < 0.002), consistent with its contractile composition. The fibrous cap was enriched for both immune cell and VSMC markers (*P* < 0.002), supporting an intermediate profile with both structural and inflammatory features.

Pathway enrichment analysis further supported the correspondence between molecular profiles and histomorphological features across subregions (Fig. 4d and Supplementary Table 6)^30^. The necrotic core was enriched for inflammation, immune activation and catabolic processes, together with platelet aggregation and fibrin clotting pathways. The media was enriched for smooth muscle contraction, muscle development and cell–ECM interaction pathways. The fibrous cap again showed an intermediate profile, sharing features of both the media and necrotic core, including ECM remodeling, VSMC differentiation and inflammatory processes.

### Comparative analysis of plaque cap thickness status

**Fig. 5:**
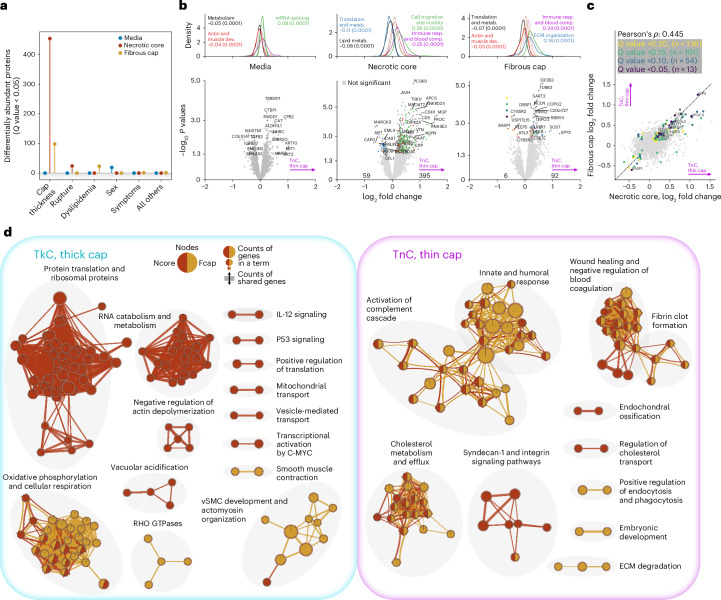
MCT status is associated with subregion-specific proteomic programs. **a**, Number of proteins significantly associated with clinical covariates in each plaque subregion (*q* < 0.05; moderated *t*-test). **b**, Protein abundance differences by MCT status within each plaque subregion (media, necrotic core and fibrous cap). Upper panels show distributions of log_2_ fold changes (TnC versus TkC) for proteins assigned to each MG, with median fold changes and bootstrap-derived *P* values indicated. Lower panels show protein-level differential abundance; numbers indicate significantly different proteins (two-sided bootstrap test, *q* < 0.05). Points are colored by MG. **c**, Concordance of MCT-associated protein abundance changes between the necrotic core and fibrous cap, shown as log_2_ fold changes (TnC versus TkC) in each subregion (Pearson’s *ρ* = 0.445). Proteins are colored based on statistical confidence (*q*-value thresholds). **d**, Network representation of enriched biological processes among proteins associated with MCT plaque phenotypes, showing distinct pathway enrichment patterns in TkC (left) and TnC (right) plaques. Nodes represent enriched terms, with size proportional to the number of proteins per term and pie charts indicating subregional contribution; edges connect related terms on the basis of shared proteins. Source data

Having established that plaque subregions are associated with distinct biological programs, we next examined the relationship between covariates, protein abundance and plaque MCT status. Association patterns varied across subregions and covariates (Fig. 5a). In the necrotic core, 454 proteins were significantly associated with MCT status, whereas 17 were associated with plaque rupture status (*q* < 0.05). By comparison, 98 proteins in the fibrous cap were associated with MCT status. Analyses involving other clinical covariates, including dyslipidemia, sex and symptom status, should be interpreted as exploratory because of class imbalance (Supplementary Table 1).

The necrotic core showed the largest proteomic differences by MCT status, with 395 proteins present at higher abundance in TnC than in TkC plaques (*q* < 0.05; Fig. 5b). These included MGP and AHSG, consistent with altered ossification-related or calcification-related processes in TnC plaques. Proteins involved in triglyceride-rich lipoprotein biology and lipid processing were also increased, including APOC3, APOA5 and PCSK9. In addition, several ECM-remodeling proteins, including GAS6, ACAN and DPT, were more abundant in TnC plaques. Immune-response-related and cell-migration-related modules were also increased in the necrotic core of TnC plaques (*P* < 0.0001; Fig. 5b).

In the fibrous cap, proteomic differences combined structural, inflammatory and matrix-remodeling features that partially overlapped with those seen in the necrotic core (Fig. 5b). TnC plaques showed higher abundance of proteins linked to ECM remodeling or destabilization, including SOST, TUBB3 and REEP5, as well as immune-associated proteins such as LRRK1 and NEK9. Consistent with this, ECM reorganization and immune-process modules were increased (*P* < 0.0001; Fig. 5b), whereas translation-associated and smooth muscle-associated modules were decreased. By contrast, the media showed comparatively modest abundance differences between MCT groups (Fig. 5b). Cytokeratins (KRT10, KRT1 and KRT2) and cell-differentiation-associated markers showed the largest shifts, which may be consistent with altered VSMC state during medial remodeling; however, these differences did not reach statistical significance (*q* > 0.05).

**Extended Data Fig. 5:**
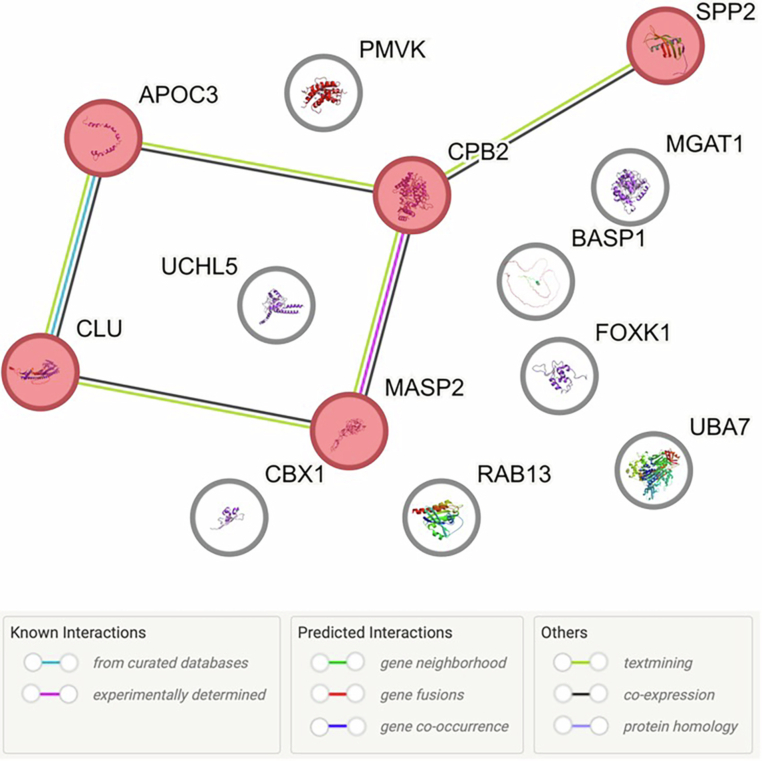
STRING network of proteins with concordant TnC-associated changes across necrotic core and fibrous cap. STRINGdb protein–protein interaction network for proteins showing correlated differential abundance between necrotic core and fibrous cap.

**Extended Data Fig. 6:**
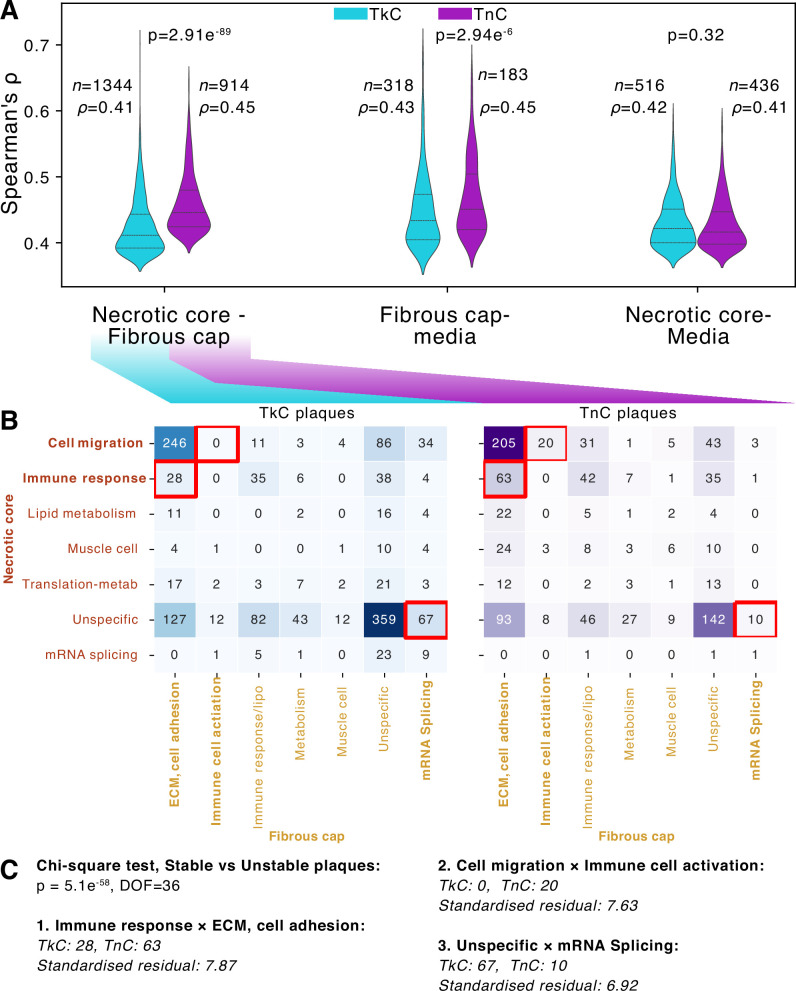
Cross-subregion protein–protein correlation architecture differs between TkC and TnC plaque phenotypes. **A**, Violin plots showing the distribution of Spearman correlation coefficients (ρ) for inter-subregion protein–protein pairs (necrotic core–fibrous cap, fibrous cap–media, and necrotic core–media), computed separately in TkC (cyan) and TnC (purple) plaques. Proteins were restricted to those associated with plaque MCT status within each subregion (necrotic core and fibrous cap: FDR q < 0.05; media: nominal P < 0.05). Only protein pairs exhibiting significant cross-subregion correlation (pairwise P < 0.005) were retained; n indicates the number of retained pairs and the median ρ is shown for each comparison. P values above violins correspond to two-sided Mann–Whitney U tests comparing the ρ distributions between TkC and TnC plaques. **B**, Contingency heatmaps summarising the number of significant necrotic core–fibrous cap correlated protein pairs, stratified by molecular-group (module) membership (rows, necrotic core; columns, fibrous cap), shown for TkC plaques (left) and TnC plaques (right). Each cell reports the count of correlated pairs linking the corresponding molecular groups (annotated by their dominant functional theme). **C**, Global comparison of the necrotic core × fibrous cap contingency tables between TkC and TnC plaques using a two-sided χ^2^ test of homogeneity (table-level test; degrees of freedom as indicated). Red-outlined cells mark the largest absolute standardised residuals, highlighting molecular-group interactions most over- or under-represented relative to expectation and contributing most strongly to the overall χ^2^ statistic.

Correlation of MCT-associated protein abundance changes between the necrotic core and fibrous cap identified 13 proteins altered in the same direction across both subregions (Pearson’s *ρ* = 0.445, *q* < 0.05; Fig. 5c). Among these, SPP2, a protein linked to calcification and matrix remodeling, showed higher abundance in both subregions. In silico protein-interaction analysis suggested a connected network involving calcification-related proteins (SPP2), thrombosis-associated regulation (CPB2), triglyceride-rich lipoprotein/remnant-associated apolipoproteins (APOC3), cytoprotective factors (CLU) and complement-associated inflammation (MASP2; Extended Data Fig. 5). We further examined spatial coordination by comparing protein–protein correlations across subregions. The necrotic core–fibrous cap comparison showed the strongest statistical evidence for a correlated change by MCT status, although the absolute difference was small (ρTkC = 0.41 versus ρTnC = 0.45, *P* = 2.91 × 10^−89^; Extended Data Fig. 6). Immune response-ECM remodeling, cell migration-immune activation and mRNA-splicing modules showed the strongest coordinated patterns between the necrotic core and fibrous cap in TnC plaques (*P* = 5.1 × 10^−58^, chi-squared test; Extended Data Fig. 6b,c), consistent with spatially patterned inflammatory and structural differences between plaque phenotypes.

Finally, integrative pathway enrichment analysis further distinguished TnC from TkC plaques (Fig. 5d and Supplementary Table 7). TkC plaques were enriched for cellular respiration pathways, including oxidative phosphorylation (*q* = 2 × 10^−4^) and aerobic respiration (*q* = 1.2 × 10^−3^), as well as protein synthesis pathways, including cytoplasmic translation and cytoplasmic ribosomal proteins (both *q* < 1 × 10^−5^), and smooth muscle cell programs, including muscle cell development (*q* = 1.1 × 10^−3^) and smooth muscle contraction (*q* < 1 × 10^−5^). These findings are consistent with greater representation of metabolic, translational and smooth muscle-associated programs in TkC (or less adverse outcome) plaques^18^. By contrast, TnC plaques were enriched for immune response, cholesterol metabolism, wound healing, ossification, ECM degradation and Syndecan-1 and integrin signaling pathways. Collectively, these analyses identify inflammation, lipid handling, altered smooth muscle-associated programs and ECM remodeling as biological processes associated with the TnC plaque phenotype^19^.

### Prioritizing PCSK9 and VSMC response to necrotic core-like stress

**Extended Data Fig. 7:**
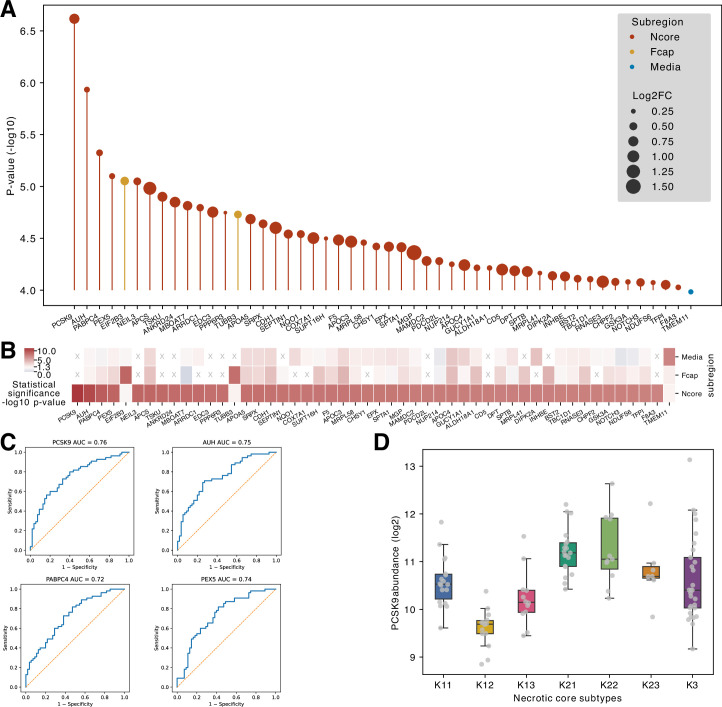
Prioritisation of PCSK9 for mechanistic follow-up based on MCT association and single-marker performance. **A**, Lollipop plot ranking proteins by their association with MCT status in subregion-resolved analyses. The y-axis shows statistical significance ( − log10 P value, moderated F-statistic test) from the MCT association test; point size reflects the absolute effect size ( | log2‌ fold change | , TnC vs TkC), and point colour indicates the plaque subregion in which the association is strongest (necrotic core, fibrous cap, or media). **B**, Subregion specificity of the top-ranked MCT status associated proteins. Heatmaps summarise effect sizes (colour scale) and statistical significance ( − log10 P value, moderated F-statistic test) across media, fibrous cap, and necrotic core. PCSK9 ranks highest overall and shows an MCT association that is predominantly confined to the necrotic core. **C**, Single-marker discrimination of TnC versus TkC plaque phenotypes using necrotic-core protein abundance. Receiver operating characteristic (ROC) curves are shown for PCSK9 and the next four top-ranked proteins (AUH, PABPC4, PEX5, EIF2B3), evaluated by logistic regression with 5-fold cross-validation. Reported AUC values are cross-validated estimates; the dashed diagonal indicates chance performance. **D**, PCSK9 abundance (log2) across necrotic core proteomic subclusters (K11, n = 18; K12, n = 13; K13, n = 12; K21, n = 19; K22, n = 11; K23, n = 7; K3, n = 28). Boxplots show the median and interquartile range (IQR); whiskers indicate 1.5×IQR; points represent individual samples. PCSK9 differs across subclusters (one-way ANOVA, P < 0.0001), with highest levels in K21 and K22.

We prioritized PCSK9 for follow-up experimental evaluation based on three observations. First, PCSK9 was among the most statistically significant proteins associated with the TnC plaque phenotype and showed higher abundance predominantly in the necrotic core (Extended Data Fig. 7a,b). Second, PCSK9 showed the strongest individual discrimination between TnC and TkC plaque phenotypes in this dataset (AUROC = 0.76; 5-fold cross-validated receiver operating characteristic (ROC); Extended Data Fig. 7c). Third, PCSK9 abundance varied across necrotic core subclusters and was highest in K21 and K22 (*P* < 0.0001, one-way analysis of variance or ANOVA), suggesting that its association with TnC status is concentrated in specific necrotic core states (Extended Data Fig. 7d).

**Fig. 6:**
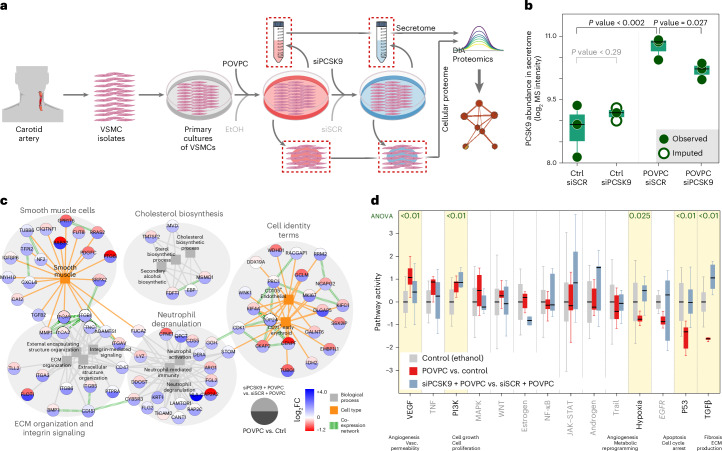
PCSK9-associated responses to oxidative/inflammatory stress in primary VSMCs. **a**, Experimental design for modeling oxidative/inflammatory stress responses in primary human VSMCs. VSMCs isolated from CEA specimens were cultured under control conditions (ethanol vehicle) or stimulated with POVPC, with parallel PCSK9 knockdown (siPCSK9) or negative-control siRNA (siSCR). Cellular proteomes and secretomes were profiled by data-independent acquisition (DIA) proteomics and integrated for downstream analyses. **b**, PCSK9 abundance in the secretome across experimental conditions (*n* = 3 per condition, *P* < 0.05). Points indicate biological replicates, boxes indicate the median and interquartile range, and whiskers show the data range. Filled circles indicate observed values, and open circles indicate imputed values; *P* values (*t*-test) for the indicated pairwise comparisons are shown. **c**, Knowledge graph summary of POVPC-associated and PCSK9-associated molecular changes. Nodes represent genes/proteins (circles), biological processes (gray squares) and cell-identity terms (orange squares); edges indicate curated term–gene links and coexpression relationships (green dashed lines). For each gene/protein node, the upper half shows the log_2_ fold change for the PCSK9 knockdown effect under POVPC stimulation (siPCSK9 + POVPC versus siSCR + POVPC), and the lower half shows the log_2_ fold change for POVPC versus control (ethanol). Color indicates direction and magnitude of change (blue, increased; red, decreased; scale shown). **d**, Pathway activity changes relative to control (ethanol; gray, *n* = 6) after POVPC stimulation (red, *n* = 9) and after PCSK9 knockdown with POVPC stimulation (blue, *n* = 9). Shaded regions indicate pathways with significant overall differences (*P* ≤ 0.025, one-way ANOVA), boxes indicate the median and interquartile range, and whiskers show the data range. Source data

To relate the necrotic-core-enriched PCSK9 signal in TnC plaques to a plausible cellular context, we tested whether oxidative and inflammatory stress resembling features of the necrotic core alters VSMC state and PCSK9 secretion. We focused on VSMCs because they can secrete PCSK9 and exhibit disease-relevant phenotypic plasticity in response to local stressors^31–36^. We hypothesized that an oxidative/inflammatory environment resembling that of TnC necrotic cores would promote a more inflammatory VSMC state and be associated with increased PCSK9 secretion. To test this, we modeled necrotic core-like stress in primary VSMC cultures using 1-palmitoyl-2-[5-oxovaleroyl]-sn-glycero-3-phosphocholine (POVPC), a pro-inflammatory oxidized phospholipid enriched in advanced atherosclerotic lesions (Fig. 6a)^37–39^. We then quantified cellular responses and PCSK9 secretion following POVPC stimulation and small interfering RNA (siRNA)-mediated PCSK9 knockdown (siPCSK9, based on the inclisiran nucleotide sequence without the *N*-acetylgalactosamine moiety)^40^.

**Extended Data Fig. 8:**
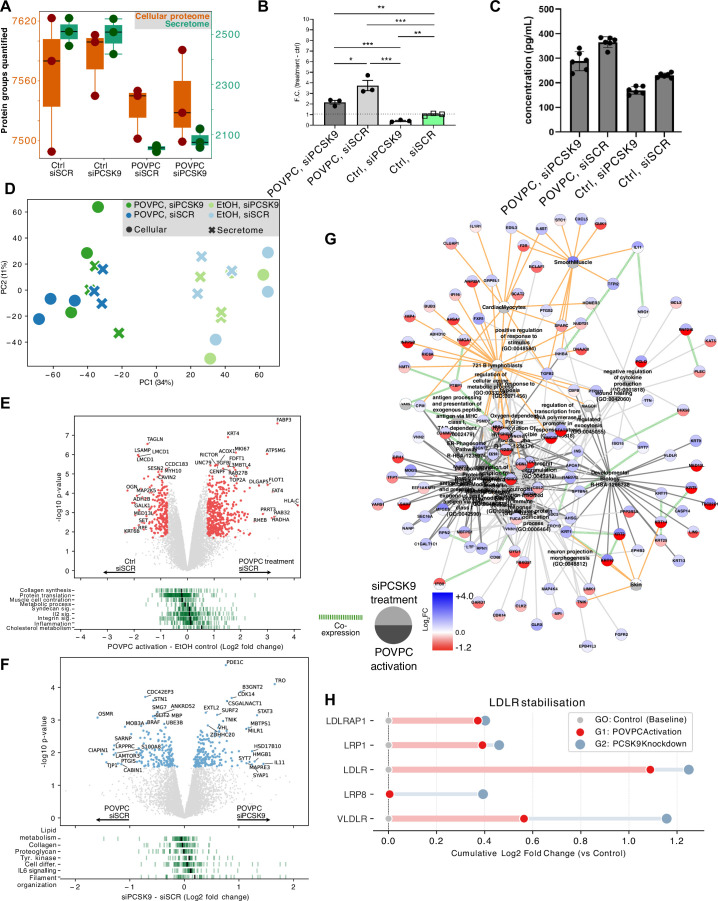
Multi-omic profiling of POVPC stimulation and PCSK9 knockdown in primary vascular smooth muscle cells. **A**, Numbers of protein groups quantified in the cellular proteome (orange; left y-axis) and secretome (green; right y-axis) across experimental conditions (Ctrl/EtOH vs POVPC; siSCR vs siPCSK9, n = 3 per condition). Points indicate biological replicates, boxes indicate median and interquartile range, with whiskers showing the data range. **B**, PCSK9 mRNA abundance measured by qPCR across conditions, shown as fold change relative to the control siSCR group (dotted line, n = 3 per condition). Horizontal brackets indicate the tested comparisons; significance (P-value < 0.05, two-sided t-test) is denoted by asterisks as shown, bars show mean with individual replicates overlaid and error bars indicate standard deviation. **C**, PCSK9 protein concentration in conditioned media quantified by ELISA across the same conditions (bars show mean with individual replicates overlaid and error bars indicate standard deviation, n = 6 per condition). **D**, Principal component analysis of paired cellular proteome (circles) and secretome (crosses) profiles, demonstrating separation by treatment and knockdown condition (colours as indicated). **E**, Differential protein abundance in the cellular proteome following POVPC stimulation (POVPC vs EtOH control; siSCR background). Red points indicate proteins significant at P < 0.05 (moderated F-statistic test); selected proteins are annotated. The lower track summarises enrichment of curated functional categories among the differential proteins. **F**, Differential protein abundance associated with PCSK9 knockdown under POVPC exposure (siPCSK9 vs siSCR in POVPC-treated cells), displayed as a volcano plot (P < 0.05 highlighted, moderated F-statistic test; selected proteins annotated), with functional-category enrichment shown below. **G**, Integrated knowledge-graph representation of the protein changes associated with PCSK9 knockdown and POVPC activation. Nodes represent proteins/genes and higher-order terms (biological processes and cell-identity terms), and edges denote curated term–gene links and co-expression relationships. For each protein node, the upper half encodes the log2 fold change for siPCSK9 vs siSCR (under POVPC), whereas the lower half encodes the log2 fold change for POVPC vs EtOH control (colour scale shown). **H**, LDLR stabilisation responses in cultured vascular smooth muscle cells across experimental conditions (control, POVPC activation, and PCSK9 knockdown). Dumbbell plots show cumulative log2 fold changes (vs control) of proteins. Grey points indicate baseline (control), red indicates POVPC activation, and blue indicates PCSK9 knockdown in POVPC activated VSMCs.

Our proteomic assays quantified an average of 7,563 cellular proteins and 2,345 secreted proteins (Extended Data Fig. 8a). PCSK9 abundance increased 1.8-fold after POVPC treatment (*P* < 0.002, two-sided *t*-test) and decreased after siPCSK9 treatment (0.5-fold relative change, *P* = 0.027; Fig. 6b). Independent quantitative PCR and enzyme-linked immunosorbent assay experiments were consistent with the mass spectrometry results (Extended Data Fig. 8b,c). POVPC treatment was the major source of proteomic variation in VSMCs, with PC1 explaining 34% of the total variance and separating samples by treatment status (Extended Data Fig. 8d). In addition, 748 proteins were increased after POVPC treatment (*q* < 0.05; Extended Data Fig. 8e). Pathway analysis indicated enrichment of neutrophil degranulation, ECM organization, integrin signaling and cholesterol metabolism in POVPC-treated VSMCs (Fig. 6c). By contrast, PCSK9 knockdown in POVPC-treated VSMCs was associated with increased cell-development-related processes, enhanced negative regulation of cytokine production and relative stabilization of LDLR abundance (Extended Data Fig. 8f–h).

**Extended Data Fig. 9:**
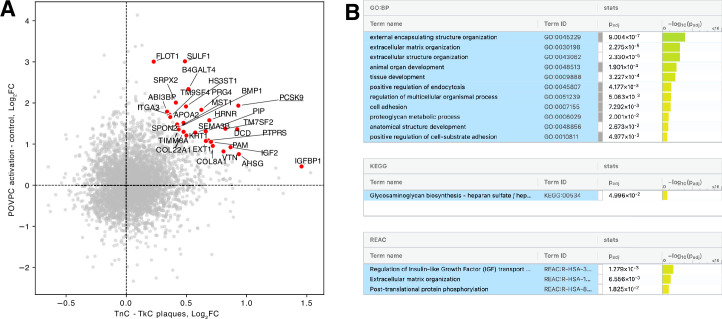
PCSK9 abundance aligns between plaque MCT status and POVPC-induced VSMC activation. **A**, Cross-system concordance between human plaque MCT status and in vitro VSMC activation. Scatterplot compares log2 fold changes for proteins quantified in necrotic core (TnC vs TkC plaques; x-axis) and in VSMCs (POVPC-treated vs control; y-axis). Each point represents a protein. Proteins concordantly upregulated in both contrasts were ranked by the geometric mean of the two log2 fold changes; the top 30 are highlighted (red) and labelled, including PCSK9. **B**, Functional enrichment analysis of the 30 concordantly upregulated proteins. Enriched terms highlight extracellular matrix/extracellular structure organisation, cell adhesion and proteoglycan metabolism (including heparan sulfate/heparin biosynthesis), and IGF transport/uptake pathways (multiple-testing–adjusted P values shown; g:Profiler output).

We next assessed pathway activity changes associated with POVPC treatment and PCSK9 knockdown (Fig. 6d)^41^. POVPC treatment was associated with increased vascular endothelial growth factor signaling and reduced P53-related and transforming growth factor-beta-related pathway activity. In contrast, PCSK9 knockdown in POVPC-treated VSMCs was associated with partial restoration of vascular endothelial growth factor, hypoxia and P53 pathway activity, together with increased PI3K-related and transforming growth factor-beta-related signaling. Overall, these findings indicate that oxidative/inflammatory stress induces broad proteomic remodeling in VSMCs and is associated with increased PCSK9 abundance and secretion. PCSK9 knockdown was associated with more modest shifts in downstream programs, including changes in cytokine-related, growth-related and ECM-related pathways. Cross-system concordance analysis further showed overlap between proteins increased in POVPC-treated VSMCs and those increased in necrotic cores of TnC plaques (Extended Data Fig. 9a). Shared signals were enriched for ECM/structure organization, cell adhesion and proteoglycan metabolism, including heparan sulfate/heparin biosynthesis, as well as insulin-like growth factor transport pathways (Extended Data Fig. 9b).

### Prioritizing proteins for predicting TnC plaque status

**Fig. 7:**
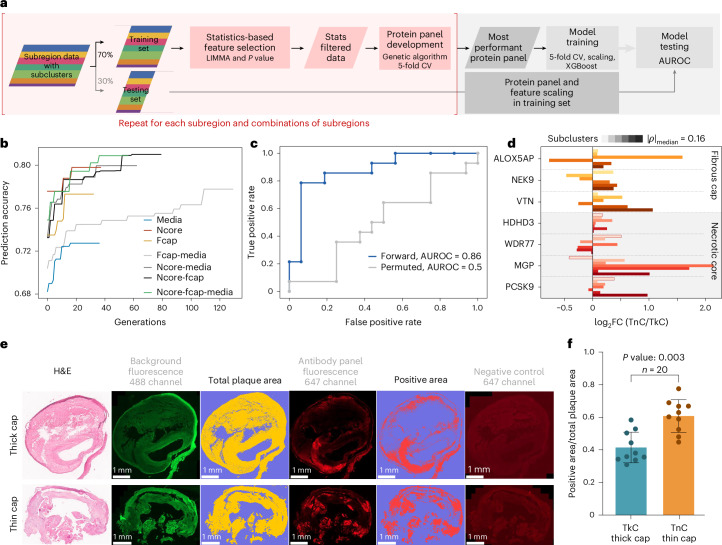
A multi-subregion protein panel discriminates TnC plaque phenotype and is supported by orthogonal tissue analysis. **a**, Machine-learning framework for deriving protein panels that discriminate MCT status from subregion proteomes. For each plaque subregion, and for combinations of subregions, data were split into training (70%) and test (30%) sets. Proteins were first pre-filtered by statistical feature selection (limma; *P*-value thresholding), followed by genetic-algorithm-based selection with 5-fold cross-validation (CV) to identify candidate panels. The best-performing panel was then used for model training, including feature scaling and 5-fold CV, and evaluated in the held-out test set by AUROC. **b**, Optimization trajectory of model performance across genetic-algorithm generations, showing improved discrimination when proteins from multiple plaque subregions were combined relative to single-subregion panels. **c**, ROC curve for the optimized protein panel (AUROC = 0.86). The gray curve shows performance under label permutation (AUROC = 0.5); significance was assessed by permutation testing (*n* = 50,000). **d**, log_2_ fold changes (TnC versus TkC) for the seven proteins in the final panel across plaque subregions. Bars are colored by necrotic core subcluster membership; the median absolute correlation is indicated (median |*ρ* | = 0.16). **e**, Multiplex immunofluorescence analysis of the panel in an independent set of human plaques. Representative sections show hematoxylin and eosin (H&E) staining, background fluorescence, segmentation of total plaque area, antibody-panel signal, the corresponding positive-area mask and a negative-control channel for TkC and TnC plaques. **f**, Quantification of panel-positive area normalized to total plaque area in the independent cohort (*n* = 10 TkC carotid plaques, 10 TnC carotid plaques), showing higher signal in TnC plaques (two-sided *t*-test, *P* = 0.003). Source data

Building on our ability to resolve histomorphology-confined differences between TkC and TnC plaques, we next evaluated whether these data could be used to derive a protein panel associated with TnC plaque status and assess its performance in an independent assay. Given the hierarchical structure of the dataset, we implemented an integrated prediction framework that incorporated both spatial information (plaque subregion) and molecular heterogeneity (subcluster assignment). This framework combined effect-size-based statistical filtering, metaheuristic optimization and ensemble learning to derive a candidate protein panel (Fig. 7a).

This approach identified a hierarchical pattern consistent with the preceding analyses. A marker panel derived from necrotic core proteins showed the highest discrimination among single-subregion models (AUROC = 0.79), followed by panels derived from the fibrous cap (AUROC = 0.77) and media (AUROC = 0.73; Fig. 7b). Combining proteins across subregions further improved discrimination (AUROC = 0.82). An optimized seven-protein panel derived from the necrotic core and fibrous cap showed the highest overall performance for discriminating TnC from TkC plaques (AUROC = 0.86, *P* < 0.0001, permutation test; Fig. 7c).

The seven-protein panel comprised proteins with diverse annotated functions relevant to plaque biology. In the fibrous cap, these included ALOX5AP, which is linked to leukotriene signaling and inflammatory responses; NEK9, which has been implicated in microtubule regulation and cellular stress responses; and VTN, which is associated with ECM organization and hemostatic processes. In the necrotic core, the panel included HDHD3, WDR77, MGP and PCSK9, encompassing proteins linked to nucleotide metabolism, transcriptional regulation, calcification-related processes and lipid metabolism.

Pairwise correlations among panel proteins were modest (median absolute Pearson’s *ρ* = 0.16), indicating limited redundancy across the selected markers (Fig. 7d). This suggests that the panel captures partially nonoverlapping information. To assess performance in an orthogonal assay, we measured the panel by quantitative immunofluorescence in an independent cohort of TkC and TnC plaques (*n* = 20; Fig. 7e) selected using criteria similar to those applied to the proteomics cohort (Supplementary Table 1). This analysis provided independent support for separation between groups in the validation cohort (*P* = 0.003, two-sided *t*-test; Fig. 7f). Together, these findings indicate that integrating subregion-resolved and subtype-aware proteomic information can improve discrimination of TnC plaque status.

### Markers of TnC plaque phenotype in serum proteomes

**Fig. 8:**
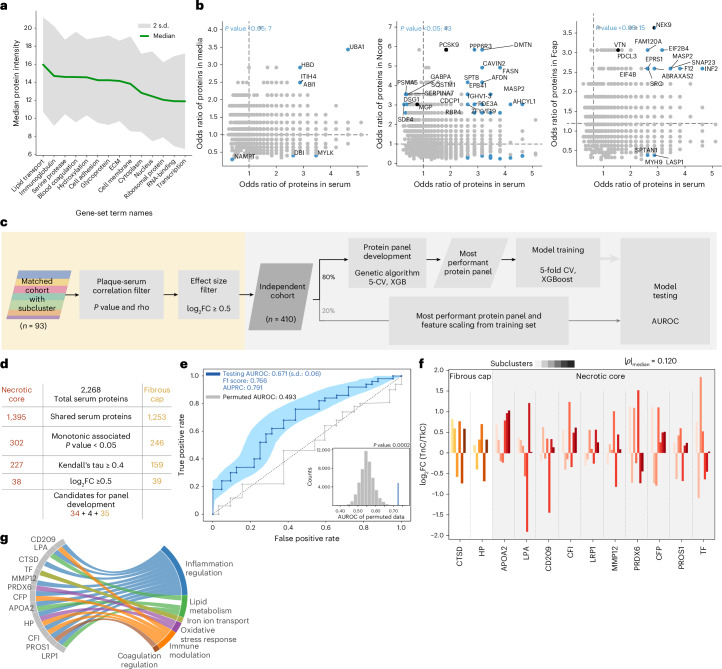
Derivation of a serum protein panel associated with TnC plaque phenotype. **a**, Global distribution of serum protein intensities across functional gene-set categories. The green line indicates median protein intensity, and the gray band denotes ±2 s.d. across all serum proteins. **b**, Concordance of TnC-associated enrichment between serum and plaque subregions. For each protein, the odds ratio for enrichment in TnC versus TkC plaques in serum (*x* axis) is plotted against the corresponding odds ratio in plaque media (left), necrotic core (middle) or fibrous cap (right). Odds ratios >1 indicate enrichment in TnC plaques. Blue points indicate proteins significant in both serum and the corresponding plaque subregion (two-sided Fisher’s exact test, *P* < 0.05; counts indicated), and black points indicate proteins from the optimized plaque tissue panel. **c**, Workflow for serum biomarker discovery. Candidate proteins were first filtered in a matched serum-plaque cohort with subcluster information (yellow; *n* = 93) using plaque-serum correlation (*P* value and *ρ*) together with an effect-size threshold ( | log_2_ fold change | ≥0.5). Candidates were then optimized in an independent serum cohort (gray; *n* = 410) using a genetic algorithm with 5-fold cross-validation and XGBoost, and performance was assessed by AUROC in a held-out validation split. **d**, Stepwise reduction of serum proteins during filtering, shown separately for necrotic-core-informed and fibrous-cap-informed candidates, including the number retained at each step and the final candidate set carried forward for panel optimization. *P* value was determined from Spearman’s correlation. **e**, ROC curve for the optimized serum panel (blue; AUROC = 0.67), with the label-permuted null model shown in gray. The inset shows the AUROC distribution from 50,000 permutations and the corresponding permutation *P* value. AUPRC, area under the precision-recall curve. **f**, TnC-associated abundance patterns of the 12 proteins in the final serum panel in the matched cohort, shown as log_2_ fold changes (TnC versus TkC). Shading indicates the corresponding plaque subcluster; the median absolute plaque-serum correlation across the panel is shown (median |*ρ* | = 0.12). **g**, Functional annotation of the 12 serum proteins (left) and their associated biological processes (right), shown as a protein–process linkage diagram. Source data

Having identified proteomic signatures associated with MCT status in plaque tissue, we next asked whether corresponding signals could also be detected in serum. We profiled serum proteomes from 505 patients undergoing CEA with TkC and TnC plaques (Supplementary Table 1) and quantified 2,268 proteins in 503 serum samples using a perchloric acid-based serum proteomics enrichment method^42^. In contrast to plaque tissue proteomes, the highest-abundance serum proteins were dominated by lipid transport proteins, immunoglobulins and serine proteases, whereas lower-abundance proteins more often included cytoplasmic, nuclear and transcription-associated proteins (Fig. 8a). This abundance gradient, from canonical serum proteins to lower-abundance proteins plausibly reflecting tissue leakage, is consistent with compartmentalization of the serum proteome^43^.

Among plaque subregions, the necrotic core showed the greatest overlap with serum-associated proteins (43 proteins, compared with 15 for the fibrous cap and 7 for the media; Fig. 8b). We then adapted our plaque-derived marker-selection framework for serum analysis to prioritize proteins associated with the TnC plaque phenotype (Fig. 8c). Initial feature selection was performed in a subset of cases with matched plaque and serum samples (*n* = 93), while the remaining 410 samples were used for panel optimization and performance assessment. We filtered the 2,268 serum proteins using monotonic associations with plaque proteins together with effect-size criteria for TnC status, which reduced the candidate set to 38 necrotic-core-associated and 39 fibrous-cap-associated proteins (Fig. 8d). Genetic-algorithm-based optimization identified a 12-protein panel with an AUROC of 0.67 (*P* = 0.0002, permutation test; Fig. 8e).

This optimized panel showed distinct subregion-related abundance patterns and subtype associations, with modest overall correlation among panel proteins (median Pearson’s *ρ* = 0.120; Fig. 8f). Functional annotation indicated representation of processes relevant to TnC-associated plaque biology, including inflammation regulation (CD209, HP, CFI), lipid metabolism (APOA2, LPA), oxidative stress responses (PRDX6, HP), iron ion transport (TF) and coagulation-related regulation (PROS1; Fig. 8g). Overall, these findings suggest that serum proteomics captures a limited but detectable signal related to plaque MCT status. However, the modest discrimination achieved here indicates that substantial further optimization would be required before a serum-based signature could support noninvasive assessment of plaque phenotype.

## Discussion

Our spatial proteomic analysis of advanced carotid plaques from patients undergoing CEA indicates that molecular programs associated with MCT are not uniformly distributed across the plaque but are concentrated within specific subregions. This links a histology-based measure of cap thickness to spatially resolved molecular patterns within the lesion.

A central finding is that MCT-associated differences were most evident in the necrotic core and, to a lesser extent, the fibrous cap. Using histology-guided microdissection and high-resolution mass spectrometry-based proteomics, we found enrichment of immune/inflammatory activation, lipid handling, ECM remodeling and ossification/calcification-related pathways in TnC plaques, particularly within the necrotic core, consistent with prior evidence linking local inflammation and lipid dysregulation to adverse plaque biology^44,45^. A protein panel derived from these spatial data discriminated TnC from TkC plaques and showed orthogonal support in an independent tissue cohort, whereas performance in serum was modest. These findings support the value of spatial context for marker prioritization, but do not yet establish clinical utility.

The fibrous cap also showed proteomic differences associated with TnC status, including inflammatory, cholesterol-related and ECM-remodeling signals. The enrichment of ECM-modulatory proteins is consistent with active local remodeling of the cap rather than a purely passive structural role, and aligns with models in which thin caps reflect interactions among inflammatory cells, VSMC plasticity and ECM turnover^46^. By comparison, the media showed fewer TnC-associated changes, although the observed signals were compatible with altered VSMC state^47–49^. More broadly, our data recapitulate molecular themes reported in bulk plaque studies while suggesting that these programs are differentially distributed across plaque subregions^16–18^.

The combined analysis of human plaque data and the in vitro VSMC model also suggests an association between the oxidative/inflammatory milieu of TnC necrotic cores, pro-inflammatory phenotypes of VSMCs and increased PCSK9 abundance. In a reductionist model, POVPC exposure induced broad VSMC proteomic changes and increased PCSK9 at both cellular transcript and secreted protein levels, supporting activated VSMCs as a plausible local contributor to plaque PCSK9. PCSK9 knockdown was associated with modest shifts in pathway activity, including reduced cytokine-related programs and relative enhancement of growth/repair-associated pathways. These experiments identify associated signaling changes, but do not establish the causal circuitry linking PCSK9 to plaque remodeling. It also remains uncertain whether current systemic PCSK9-directed therapies sufficiently modulate arterial PCSK9, or whether such modulation would alter plaque morphology or clinical outcomes^50^.

While spatial tissue signals are not directly deployable as circulating biomarkers, we translated these data into a surrogate protein panel by integrating plaque subregion and molecular subtype information. This panel showed good discrimination in tissue but substantially weaker performance in serum, which is consistent with dilution of plaque-derived signals in circulation, confounding by dominant systemic protein sources and interindividual variability in comorbidity and treatment. These considerations emphasize the challenge of moving from tissue-associated biology to blood-based classification.

Several limitations surround our interpretations. First, the study is restricted to advanced carotid plaques and is therefore unlikely to capture molecular processes specific to earlier lesion development, including neo-intima formation^51^. Second, the cross-sectional design provides a single time point and cannot determine whether the observed proteomic patterns predict future rupture or clinical events. Third, we selected MCT rather than symptomatic presentation as the primary endpoint. This choice reduces clinical heterogeneity but also means that the results should be interpreted as molecular correlates of fibrous-cap thickness, not direct markers of plaque vulnerability or cerebrovascular risk^4,52,53^. In addition, hemorrhage-associated biology is likely to be underrepresented because overtly blood-rich regions were intentionally excluded during microdissection.

Our in vitro data support further investigation of PCSK9-related signaling but remain mechanistically preliminary. More detailed perturbational work, including phosphoproteomic and protein-interaction approaches, will be required to define causal pathways^35,54–56^. More generally, discovery proteomics may incompletely capture low-abundance components of canonical inflammatory and lipid pathways, and some signals detected in plaques may still reflect contributions from circulating proteins. For these reasons, pathway-level and module-level shifts are likely to be more robust than inference from any single marker.

Within these constraints, our data identify lipid handling, ECM remodeling, inflammation and ossification/calcification as molecular programs associated with the MCT-defined TnC phenotype, with the strongest signals in the necrotic core and fibrous cap. The observation that individual subregions contain distinct molecular subtypes further highlights the biological heterogeneity of advanced carotid plaques.

## Methods

### Ethics approval

Informed written consent for biobank sample contributions was obtained from all participants in accordance with the principles of the Declaration of Helsinki. The study protocol was approved by the local ethics committee of the Technical University of Munich (DigiMed ethics 2023-297; approval no. 2799/10). Clinical data were retrieved from anonymized electronic patient records.

### Sample collection and processing for biobank

#### Plaque tissue

Carotid plaques were obtained from patients undergoing CEA. Immediately after surgical retrieval, plaque samples were transferred into RNAlater (Thermo Scientific, Germany) to preserve RNA during transport and subsequent processing. Plaques were fixed in 4% paraformaldehyde for 24 h and decalcified with Entkalker soft SOLVAGREEN (Carl ROTH) for 5 days. Following decalcification, plaque tissues were embedded in paraffin and stored as FFPE blocks.

#### Serum samples

Blood was collected from patients undergoing CEA using S-Monovettes (Starstedt). All collected blood samples were allowed to clot at room temperature for 15 min. Subsequently, the clotted blood was centrifuged at 2,000*g* at 4 °C for 10 min, and the supernatant serum was obtained and stored at −80 °C for further processing.

### Cohort design

From a total cohort of 524 patients, carotid plaque tissue was available from 132 patients and serum was available from 505 patients. Plaque tissue from 112 patients was used for spatial proteomic analysis, whereas plaque tissue from the remaining 20 patients was reserved for immunofluorescence validation. Serum proteomics was performed in all 505 patients with available serum samples. Among the proteomics cohorts, 93 patients had matched plaque and serum samples available for paired analyses (Supplementary Table 1).

Plaque samples (*n* = 112 for proteomics; *n* = 20 for immunofluorescence) were selected based on the histological availability of the fibrous cap and medial layer, along with comprehensive clinical data. A total of 505 serum samples were included, selected based on sufficient volume, no prior freeze–thaw exposure, low hemolysis and complete accompanying clinical data. Case selection was optimized to maintain a covariate balance between TkC and TnC plaque patient cohorts.

### Histomorphology-guided spatial proteomics of plaque tissues

#### Histological analysis

FFPE plaque specimens were sectioned at 2-μm thickness and mounted on glass slides (Menzel SuperFrost, 76 × 26 × 1 mm, Fisher Scientific). Following deparaffinization and rehydration, sections were stained using H&E (ethanolic eosin Y solution and Mayer’s acidic hemalum solution, Waldeck) or Elastica van Gieson (picrofuchsin solution (Romeis 16th edition) and Weigert’s solution I (Romeis 15th edition)) according to the manufacturer’s protocols. Slides were coverslipped with Pertex (Histolab Products) and glass coverslips (24 × 50 mm, Engelbrecht). The H&E-stained slides were digitally scanned and used for subregion annotation.

A pathologist annotated the digital scans of the tissues to highlight three key subregions—media, fibrous cap and necrotic core—using established morphological criteria. The media was identified as the arterial wall layer composed mainly of smooth muscle cells and elastic fibers. The necrotic core was characterized per AHA guidelines and noted for its lipid-rich content, cellular debris, calcification and areas of intraplaque hemorrhage^22^. Minimum thickness of the fibrous cap served as a measure of plaque MCT status; caps measuring ≥200 μm were classified as thick caps (TkC), whereas those <200 μm were classified as thin caps (TnC)^20^.

#### Laser microdissection slide preparation

Two-micrometer PEN membrane slides (MicroDissect) were exposed to ultraviolet light at 254 nm for 1 h and then coated with Vectabond (Vector Laboratories; SP-1800-7) according to the manufacturer’s protocol. Next, two consecutive 5‑μm‑thick FFPE sections from each block were mounted on the pretreated slides and dried overnight at 37 °C. In parallel, two additional 7‑μm‑thick sections were also placed on the same type of pretreated PEN membrane slides and dried under the same conditions.

After drying, slides were heated to 56 °C for 20 min to soften the paraffin. This was followed by deparaffinization and dehydration using xylene (2 × 2 min) and a graded ethanol series (100%, 90%, 75% and 50% ethanol, each for 2 × 1 min). The slides were then air-dried briefly, ensuring the tissue was fully prepared for subsequent laser microdissection.

#### Laser microdissection

Digitally annotated H&E scans of each plaque were used as a reference and aligned with the corresponding FFPE sections on a Leica LMD7 laser microdissection microscope (Leica Microsystems). Within the annotated subregions, tissue areas with minimal intraplaque hemorrhage were prioritized for microdissection. For each subregion, 25 circles (area of 14,000 μm^2^ each) were excised from two consecutive microtome sections and collected directly into the wells of a low-binding 96-well plate positioned beneath the slides. The pooling of microdissection shapes was performed to suppress local heterogeneity within each histological subregion while preserving subregion consistency. Laser cutting was performed with a power of 57, aperture of 1–2, speed of 23 and a middle-pulse setting. The plates were sealed with adhesive foil (Covaris), centrifuged at 1,000*g* for 2 min and stored at −20 °C until further processing.

#### Sample preparation for mass spectrometry

All 336 samples were processed in a single batch to minimize potential batch effects. A semiautomated, high-throughput sample preparation method was used with a Bravo pipetting robot (Agilent). First, each well was washed with 50 μl of 100% acetonitrile and dried in a SpeedVac at 25 °C for 30 min. Next, 40 μl of 60 mM triethylammonium bicarbonate (Sigma) in MS-grade water was added to each well. The plates were then sealed with two layers of adhesive foil and heated at 95 °C for 60 min in a 96-well thermal cycler (Eppendorf).

After heating, 10 μl of 60% acetonitrile in 60 mM triethylammonium bicarbonate (resulting in a final acetonitrile concentration of 12.5%) was added, followed by heating at 75 °C for 60 min. The samples were then pre-digested with 40 ng LysC for 4 h at 37 °C, followed by overnight digestion with 60 ng trypsin in the same 96-well thermal cycler. After approximately 16 h, digestion was quenched by adding 15 μl of 6% trifluoroacetic acid to achieve a final trifluoroacetic acid concentration of 1%. Peptides were desalted using the iST 96x kit (PreOmics) according to the manufacturer’s instructions, dried in a SpeedVac, and stored at −20 °C until further analysis by mass spectrometry.

### LC–MS/MS data acquisition

Dried peptide samples were reconstituted in 6 μl of mass spectrometry loading buffer (2% acetonitrile (vol/vol), 0.1% trifluoroacetic acid (vol/vol) in MS-grade H_2_O). Given that plaque samples were stratified into subregions and distributed across four plates, a custom Python script was used to track and randomize sample strata. The samples were transferred into new plates using a custom protocol on the OT-2 (OpenTrons) liquid handling system.

An EASY-nLC 1200 (Thermo Fisher Scientific) was coupled to a timsTOF Ultra mass spectrometer (Bruker) via a nanoelectrospray ion source (CaptiveSpray, Bruker). Peptides were separated on a 50-cm in-house-packed nano-HPLC column (75-μm inner diameter) containing 1.9 μm ReproSil-Pur C18-AQ silica beads (Dr. Maisch). The column was maintained at 60 °C in a custom-built column oven. A 120-min linear gradient was delivered at a flow rate of 300 nl min^−1^, starting from 3% to 30% buffer B over 95 min, increasing to 60% buffer B over 5 min, washing at 95% buffer B for 10 min and re-equilibrating at 5% buffer B for 10 min. Buffer A consisted of 0.1% formic acid in 99.9% double-distilled water, whereas buffer B consisted of 0.1% formic acid, 80% acetonitrile and 19.9% double-distilled water.

The timsTOF mass spectrometer was operated in dia-PASEF mode with DIA windows customized based on pilot experiments using plaque samples. The DIA windows covered an *m/z* range of 350–1,200 and an ion mobility range of 0.7–1.3 cm^2^ V^−1^ s^−1^. All other parameters were set to default as recommended^21^.

### Bioinformatic preparation of the proteomics data

#### LC–MS/MS data analysis

Peptide and protein identification was performed using a library-free search in DIA-NN, using the UniProt FASTA database (Canonical, version 2022-07-27, 20,420 sequences)^57^. Raw mass spectrometry files from each plaque subregion were processed separately. Methionine oxidation was set as a variable modification, allowing up to one missed cleavage. The precursor charge range was specified from 2+ to 4+, with a precursor mass range of 350–1,200 *m/z* and peptide lengths of 7–35 amino acids. Both mass and MS1 accuracies were set to 15 based on prior estimations. Isotopolog detection and match-between-runs were enabled, and the neural network classifier was configured for single-pass mode. The ‘--relaxed-prot-inf’ option enabled more conservative protein grouping, with protein inference performed directly from the FASTA file. Library generation was performed using ‘Smart profiling’, cross-run normalization was set to ‘RT-dependent’ and the quantification strategy was ‘Robust LC (high precision)’.

#### Refined protein quantification and filtering

Peptide quantification data were used to requantify protein groups with at least five peptides. For these protein groups, pairwise correlation distances among its constituent peptides were calculated using a weighted Pearson correlation approach, chosen for its robustness against spurious correlations. The weight vector (w) was derived from a bivariate Gaussian kernel density estimate implemented in Python (scipy.stats.gaussian_kde), according to equation (1):

1 $${d}_{\mathrm{corr}}=1-\,\frac{{\sum }_{i=1}^{n}\left[{w}_{i}\left({x}_{i}-\bar{x}\right)\left({y}_{i}-\bar{y}\right)\right]}{\sqrt{{\sum }_{i=1}^{n}{w}_{i}{\left({x}_{i}-\bar{x}\right)}^{2}\,\cdot {\sum }_{i=1}^{n}{w}_{i}{\left({y}_{i}-\bar{y}\right)}^{2}}}$$

To identify a set of five highly correlated peptides per protein, agglomerative hierarchical clustering with average linkage was applied, followed by dynamic tree cutting within a distance range of 0.05–0.81. The peptides in each cluster were selected to represent the protein, and final protein intensities were recalculated using the MaxLFQ algorithm.

#### Protein and patient filtering and missing value imputation

Proteins with more than 66% missing values and samples with fewer than 1,500 quantified proteins were excluded from further analysis. Missing values were imputed using a Python-based adaptation of the multiple imputation by chained equations approach^58^, implemented via IterativeImputer in scikit-learn. Several regression estimators were evaluated, including *k*-nearest neighbors, extra-trees and random forests. Imputation performance was assessed by comparing the Frobenius norm of the correlation matrices derived from each imputed dataset against the unimputed reference matrix. Random forest-based imputation consistently outperformed other methods and was used for all subsequent analyses.

### Prediction plaque MCT status from the clinical metadata

To predict TnC plaque phenotypes from clinical data, binary and nominal variables (for example, sex, symptomatic status, diabetes) were one-hot encoded, whereas histological variables were treated as ordinal features. Continuous features (for example, age, stenosis, BMI) were transformed into seven quantile bins. This feature transformation reduces the likelihood of overfitting and artificial splitting from high-cardinality variables.

For the plaque cohort (*n* = 112), clinical metadata were randomly partitioned into a training set (75%) and a test set (25%). Model hyperparameter optimization was performed via Bayesian optimization on 5-fold cross-validation of the training set. Three different algorithms were tuned: random forest (n_estimators, max_depth, min_samples_split, min_samples_leaf, max_features), XGBoost (colsample_bytree, eta, gamma, max_depth, min_child_weight, n_estimators) and HistGradientBoost (learning_rate, max_iter, max_leaf_nodes, max_depth, min_samples_leaf).

The optimized models were evaluated on the plaque test set (*n* = 28) and an independent serum cohort (*n* = 410), with performance measured as the AUROC curve.

### Clustering of proteome data

#### Patient clusters

Recursive consensus clustering was performed on patients using the top 66% of the most variable proteins^59^. A total of 100,000 iterations of consensus clustering were conducted with the following parameters: top_value_method = ATC, partition_method = skmeans, mean_silhouette_cutoff = 0.9, min_samples = 0.15, sample_by = row and p_sampling = 0.8. AMI was used to assess the association between clinical covariates and the resulting plaque classifications. Statistical significance was evaluated via a permutation test (*n* = 500,000) using AMI as the test statistic.

#### Protein clusters

Weighted gene correlation network analysis was performed on the full proteomic dataset to identify protein coexpression modules^60^. The power parameter (ranging from 1 to 20) for soft-thresholding was optimized to approximate a scale-free topology with a degree of independence of 0.85. A signed correlation was used in the adjacency matrix to capture both positive and negative correlations. The minimum module size was set to 50 proteins, and modules were merged if their distance height was less than 0.25.

#### Overrepresentation analysis

Enrichment of biological processes in the module genes was tested using g:Profiler, using the following parameters: significant only, GO:BP, GO:CC, KEGG, REAC databases, and significance threshold set to FDR)^61^. The *P* value was determined using a hypergeometric test and was adjusted using Benjamini–Hochberg FDR. The seven most significant term names were selected and simplified into keywords using Sonnet 3.5 large language model (Anthropic).

### Comparative statistical analysis

#### Binary comparisons

All binary comparative analysis (such comparison of subregions, TnC versus TkC, and POVPC and PCSK9 knockdown) were performed using the LIMMA package in R, called into Python.

#### Multiple-testing correction

Statistical significance is reported with correction for multiple-hypothesis testing. Nominal *P* values are reported as *P*. Statistical significance adjusted using the Benjamini–Hochberg procedure is denoted as the false discovery rate (FDR), whereas *Q* indicates *P* values adjusted using the Storey procedure^62^. Finally, FDR_IHW_ denotes *P*-value correction via independent hypothesis weighting, with the median intensity of each protein group serving as the covariate^63^.

### Enrichment map analysis

Pathways were defined using the gene-set file Human_GOBP_AllPathways_no_GO_iea_June_03_2023_symbol.gmt^30^. Gene-set enrichment analysis used log_2_ fold change as the ranking metric, restricting the gene-set size to 10 and 500 (ref. ^27^). A total of 5,000 permutations were performed. Enriched pathways were visualized using the EnrichmentMap app in Cytoscape^64^. Network maps were generated for nodes with FDR *q* < 0.01 and *P* < 0.0001; nodes sharing gene overlaps with a Jaccard coefficient > 0.25 were linked by a green edge. Clusters of related pathways were identified and annotated using a Markov Cluster algorithm (AutoAnnotate v1.2), which groups pathways based on shared keywords in the pathway description. The resulting pathway clusters are denoted as major pathways and displayed in a group.

### VSMC proteomes

Human carotid artery smooth muscle cells were purchased from PeloBiotech (PB-3514-05a, lot no. 3003) and maintained in VSMC Medium (Smooth Muscle Cell Growth Medium, PB-MH-200-2100, PeloBiotech) until further use.

#### Cell culture and treatment

Human VSMCs were seeded at a density of 15,000 cells per well in six-well plates using a standard VSMC medium. After 24 h, the medium was discarded, and the cells were washed three times with phosphate-buffered saline, followed by three washes with serum-free, phenol red-free medium (SF&PRF; PB‑MH‑200‑2190‑FCS‑PRF, PeloBiotech). Cells were treated with 10 μg ml^−1^ POVPC or an equivalent volume of ethanol (solvent control) in SF&PRF medium for 72 h.

At the end of the 72-h treatment, the culture supernatant was collected (see ‘Cell harvesting’ protocol). Subsequently, 25 nM inclisiran (HY‑132591, MedChemExpress) or 25 nM negative-control siRNA (AM4614, Ambion) was introduced in SF&PRF medium using RNAiMAX (13778‑150, Thermo Fisher). Following an additional 24-h incubation, the supernatant and the cells were harvested and prepared for proteomic analysis.

#### Supernatant collection

Cell culture supernatant was transferred into 2‑ml microcentrifuge tubes and centrifuged at 300*g* for 10 min at 4 °C to remove non-adherent cells. Approximately 95% of the supernatant was transferred to a fresh 2‑ml tube and centrifuged at 2,000g for 20 min at 4 °C to remove debris. Finally, ~95% of the clarified supernatant was transferred to another 2‑ml tube and stored at −80 °C.

#### Cell harvesting

After removing the medium, cells were scraped in 1 ml of ice-cold TBS and transferred to a microcentrifuge tube. The cell suspension was centrifuged at 300*g* for 10 min at 4 °C, and the supernatant was discarded. The cell pellet was then washed with ice-cold TBS and centrifuged. This washing step was repeated twice more for a total of three washes. After the final wash, the supernatant was carefully removed, and the pellet was stored at −80 °C with minimal residual liquid.

#### Processing of cellular and secretomes for LC–MS/MS

Cell pellets were processed using the iST kit (PreOmics, Germany) following the manufacturer’s instructions. Briefly, cells were resuspended in 100 μl of the ‘lyse’ buffer and lysed using a BeatBox magnetic homogenizer (PreOmics) for 20 min at the standard setting. Lysates were denatured by heating at 95 °C for 10 min, followed by 5 min of sonication (ten cycles of 30 s on/off) to shear DNA. Proteins were digested overnight using a Trypsin–LysC mixture. Peptides were desalted with the kit’s cartridges, dried and reconstituted in 0.1% formic acid in water.

Secretome samples were thawed and concentrated to 100 μl using a 7-kDa molecular-weight-cutoff spin filter. An equal volume of lyse buffer was added to each sample, and the same denaturation, digestion and desalting steps described above were applied.

#### LC–MS/MS analysis

Samples were loaded onto Evotip Pure tips (Evosep) and separated using an Evosep One system using the standardized Whisper 40-sample-per-day (40SPD) method. Peptides were passed through a 15-cm column (75 μm inner diameter) packed with 1.7-μm C18 beads (IonOpticks), heated to 50 °C and then introduced into a timsTOF mass spectrometer (Bruker). Data acquisition settings followed previously described protocols, along with protein filtering and imputation.

#### Knowledge graph analysis

The top 250 upregulated genes were used for the knowledge graph analysis^41^. Enrichment was performed against the GO:BP and the Human Gene Atlas database. Only the top 14 terms from each database were exported.

#### Pathway activation analysis

PROGENy analysis was used to assess changes in cellular signaling pathway activity in VSMCs under three conditions: control, POVPC treatment and PCSK9 knockdown with POVPC^65^. Specifically, log_2_ fold change values (control versus control, POVPC versus control and (PCSK9 knockdown + POVPC) versus POVPC) were derived from the proteomic data. The top 50 genes for each pathway model were used in the PROGENy inference. An ANOVA test was then applied to compare pathway activity distributions across the three experimental conditions.

### Protein panel development for TnC plaque phenotype prediction

#### Feature selection

A multistep pipeline was devised to identify an optimal protein panel for predicting plaque MCT status using proteomic data from distinct plaque subregions (media, fibrous cap, necrotic core) and their combinations. First, proteomic profiles were stratified into training (70%) and testing (30%) sets, repeating this partitioning for each subregion independently and for all subregions combined. Potential features were initially selected by filtering for nominal *P* values (using the Limma framework) and consistent upregulation in TnC plaques, generating a ‘stats-filtered’ set.

#### Protein panel development

To determine the optimal panel size, 5,000 random protein subsets for each candidate size (3–20 proteins) were evaluated using an XGBoost model, resulting in a total of 90,000 AUROC evaluations. The panel size at which the AUC plateaued was deemed optimal; for plaque data, this inflection point occurred at seven proteins.

A genetic algorithm then optimized the specific composition of these seven proteins. Each generation included 6,000 candidate solutions, of which the top 2,000 were selected for mating. Four elite solutions were carried over unchanged, while mutation and crossover probabilities were initially set to 0.3 and 0.1, respectively, and rose to 0.8 and 0.6 after five generations of stagnant fitness. Within each generation, 5-fold cross-validation was performed using an Extra Trees Classifier, and optimization ceased after 20 consecutive generations without improvements in accuracy.

#### Protein panel validation

Finally, the best-performing seven-protein panel was validated with an XGBoost classifier whose hyperparameters—colsample_bytree, eta, gamma, max_depth, min_child_weight and n_estimators—were tuned via Bayesian optimization on 5-fold cross-validation of the training data.

### Quantitative immunofluorescence

#### Immunofluorescence staining

Sections were mounted on SuperFrost Plus slides (Thermo Fisher Scientific) precoated with 0.1% poly-L-lysine (Sigma-Aldrich). Antigen retrieval was performed by boiling the slides in a pressure cooker with 10 mM citrate buffer (pH 6.0), and endogenous peroxidase activity was blocked using 3% hydrogen peroxide. Sections were then blocked for 1 h with blocking buffer (5% horse serum, 1% BSA, 0.5% Triton X-100) and incubated overnight with primary antibodies diluted in 5% horse serum. After primary incubation, appropriate secondary antibodies were applied for 1 h under identical blocking conditions. Autofluorescence quenching and nuclear staining with DAPI were then performed. Primary antibodies were validated individually and combined for panel validation, and proper controls were included for each target (Supplementary Table 8). Images were acquired using a Zeiss Axioscan 7 (Carl Zeiss Microscopy) and processed with ZEN 3.3 software.

#### Quantitative analysis

Fluorescence images were analyzed using QuPath^66^. First, autofluorescence in the 488-nm channel was inspected to delineate the entire plaque area. Next, a pixel classifier threshold was applied to the 647-nm channel to capture the antibody-specific signal from the biomarker panel, enabling accurate quantification of the stained region. All subsequent calculations and statistical analyses were performed using GraphPad Prism (v10, GraphPad Software).

### Serum proteome data

#### Sample preparation

Serum samples were processed with perchloric acid as described in the original publication with modifications for automated processing^42,67^. Briefly, 5 μl of serum was diluted with 20 μl of water, followed by 25 μl of perchloric acid. The mixture was vigorously mixed for 1 h at 4 °C, then centrifuged at 4,000*g* for 20 min at 4 °C. The supernatant was transferred to μSPE hydrophilic–lipophilic balance plates (Waters) and rebuffered into 50 mM ammonium bicarbonate. Proteins were digested into peptides using trypsin and LysC (1:100 enzyme-to-protein ratio). The resulting peptides were loaded onto Evotips for desalting and LC–MS/MS analysis. All liquid handling processing steps (aliquoting, pipetting, solution transfer and rebuffering) were performed using Bravo liquid handling robot (Agilent).

#### LC–MS/MS analysis

Peptides were separated on an EvoSep One system (Evosep) using the standard 60-samples-per-day (60SPD) method, which uses a 21-min gradient. A 15-cm column (75 μm inner diameter) packed with 1.7-μm C18 beads (IonOpticks) was maintained at 50 °C and coupled to an Orbitrap Astral mass spectrometer (Thermo Fisher Scientific) via an EASY-Spray source. The spray voltage was set to 1,900 V. All samples were acquired in DIA mode at a resolution of 240,000, scanning from 380 *m/z* to 980 *m/z*. The normalized automatic gain control target was 500%, and an isolation window of 8 Th with a maximum injection time of 16 ms was used. Ions were fragmented at a higher-energy collisional dissociation collision energy of 25%. Field asymmetric ion mobility spectrometry was enabled at a compensation voltage of −40, and the gas flow was reduced to 2.5 l min^−1^.

#### Data analysis

DIA data were processed using DIA-NN as previously described. Peptides detected in fewer than 25% of samples were excluded, as were any samples with fewer than 6,000 quantified peptides. Protein groups were requantified using the correlation distance-based approach outlined earlier, and missing values were imputed using a random forest regressor in a multiple-imputation framework.

### Serum biomarker panel development

A similar multistep workflow was used to develop a serum biomarker panel, with a few modifications compared to the plaque-based approach. Serum samples were divided into two cohorts: matched (*n* = 93; samples with corresponding plaque data) and independent (*n* = 410). The matched cohort was used for initial feature selection, focusing on statistical significance, log_2_ fold change (TkC versus TnC) and monotonic correlation of protein abundance between tissue and serum. The resulting set of proteins was then used to build a biomarker panel in the independent cohort, which was further split into training and testing sets.

The optimal panel size, determined to be 12 proteins, was identified via an information-content analysis similar to that performed for plaque tissues. Panel optimization proceeded via a genetic algorithm, and the final model performance was validated in the test set.

### Statistics and reproducibility

A prior proof-of-concept study suggested a minimum sample size of 32 cases per condition. For this study, sample sizes were determined by the availability of biobank material fulfilling the predefined inclusion and quality-control criteria, including histological suitability of plaque tissue, availability of matched clinical data, sufficient serum volume, absence of prior thawing and low hemolysis. A total of 112 plaque samples were used for spatial plaque proteomics, 20 plaque samples were reserved for immunofluorescence validation, and a subset of 93 patients had matched plaque and serum proteomic data.

Data were excluded according to predefined technical and quality-control criteria. Plaque samples were selected based on histological availability of the fibrous cap and medial layer and availability of comprehensive clinical data. Serum samples were selected based on sufficient sample volume, no previous freeze–thaw cycle, low hemolysis and complete clinical data. For plaque proteomics, proteins with fewer than 27 observations and samples with fewer than 1,500 quantified proteins were excluded from downstream analyses. For serum proteomics, peptides detected in fewer than 25% of samples and samples with fewer than 6,000 quantified peptides were excluded. Missing values were imputed using a multiple-imputation framework with random forest regression, selected after benchmarking against alternative estimators.

To reduce technical bias during mass spectrometry acquisition, plaque samples were tracked and randomized across plates and acquisition order. For machine-learning analyses, samples were randomly partitioned into training and test sets as described for each analysis. Case selection for plaque proteomics was optimized to maintain covariate balance between thin-cap and thick-cap plaque cohorts. The investigators were only blinded to allocation of the MCT status of plaques.

### Reporting summary

Further information on research design is available in the Nature Portfolio Reporting Summary linked to this article.

## Supplementary information

Reporting Summary

Supplementary Tables 1–9 Supplementary Table 1. Overview of the clinical covariates for the three cohorts used in the study. Supplementary Table 2. Quantitative proteomics data matrix. Supplementary Table 3. Functional enrichment of ontology terms in module groups in the necrotic core. Supplementary Table 4. Functional enrichment of ontology terms in module groups in the media. Supplementary Table 5. Functional enrichment of ontology terms in module groups in the fibrous cap. Supplementary Table 6. Functional enrichment of ontology terms in various subregion datasets. Supplementary Table 7. Functional enrichment of ontology terms associated with MCT status across the subregions. Supplementary Table 8. Properties of antibodies used for independent validation of plaque tissue panel. Supplementary Table 9. List of proteins assigned to the MG5 module group in the necrotic core.

## Source data

Source Data Fig. 1 Statistical source data.

Source Data Fig. 2 Statistical source data.

Source Data Fig. 3 Statistical source data.

Source Data Fig. 4 Statistical source data.

Source Data Fig. 5 Statistical source data.

Source Data Fig. 6 Statistical source data.

Source Data Fig. 7 Immunofluorescence images of TnC and TkC plaques obtained using protein panel.

Source Data Fig. 8 Statistical source data.

## Data Availability

The raw mass spectrometry and search result data are accessible in the MASSIVE proteomics data repository under accession code MSV000097042.
