# Supplementary material for: Proteomics reveals spatial and molecular heterogeneities in advanced atherosclerotic carotid artery plaques
Source: Nat Cardiovasc Res. 2026 Jun 22;5(7):605–23. doi: 10.1038/s44161-026-00827-1 (PMC13368601; doi:10.1038/s44161-026-00827-1)
Supplement: Supplementary file 1 — Reporting Summary [file 44161_2026_827_MOESM1_ESM.pdf]

nature portfolio

Corresponding author(s): Matthias Mann, Lars Maegdefessel

Last updated by author(s): May 13, 2026

Reporting Summary

Nature Portfolio wishes to improve the reproducibility of the work that we publish. This form provides structure for consistency and transparency in reporting. For further information on Nature Portfolio policies, see our Editorial Policies and the Editorial Policy Checklist.

Statistics

For all statistical analyses, confirm that the following items are present in the figure legend, table legend, main text, or Methods section.

n/a	Confirmed	
☐	☒	The exact sample size (*n*) for each experimental group/condition, given as a discrete number and unit of measurement
☐	☒	A statement on whether measurements were taken from distinct samples or whether the same sample was measured repeatedly
☐	☒	The statistical test(s) used AND whether they are one- or two-sided<br>*Only common tests should be described solely by name; describe more complex techniques in the Methods section.*
☐	☒	A description of all covariates tested
☐	☒	A description of any assumptions or corrections, such as tests of normality and adjustment for multiple comparisons
☐	☒	A full description of the statistical parameters including central tendency (e.g. means) or other basic estimates (e.g. regression coefficient) AND variation (e.g. standard deviation) or associated estimates of uncertainty (e.g. confidence intervals)
☐	☒	For null hypothesis testing, the test statistic (e.g. F, t, r) with confidence intervals, effect sizes, degrees of freedom and P value noted<br>*Give P values as exact values whenever suitable.*
☒	☐	For Bayesian analysis, information on the choice of priors and Markov chain Monte Carlo settings
☐	☒	For hierarchical and complex designs, identification of the appropriate level for tests and full reporting of outcomes
☐	☒	Estimates of effect sizes (e.g. Cohen's *d*, Pearson's *r*), indicating how they were calculated

Our web collection on statistics for biologists contains articles on many of the points above.

Software and code

Policy information about availability of computer code

Data collection	Leica LMD (v8.3), Bruker HyStar (v6.0), timsControl (v3.0), Evosep One Software (RCNet Driver 2.2), Orbitrap Astral Tune Application (1.0), Thermo Scientific Xcalibur (v4.7), Zen (v3.3)
Data analysis	DIA-NN (v1.8.0), QuPath 0.4.1, GraphPad Prism (v10), Enrichr-KG (Online), GSEA (v4.3.3), Cytoscape (v3.10), EnrichmentMap (v3.5), Python (v3.11), R (v4.4), R-packages: Limma (v3.60), Cola (v2.10), ComplexHeatmap (v2.13), WGCNA (v.1.7)<br>Python-packages: SciPy (v1.13.1), Matplotlib (v3.8.4), Pandas (v2.2.2), Numpy (v1,26), PyGAD (v1.2.3), Scikit-learn (v1.4.2), PROGENy (v1.17), XGBoost (v2.0)

For manuscripts utilizing custom algorithms or software that are central to the research but not yet described in published literature, software must be made available to editors and reviewers. We strongly encourage code deposition in a community repository (e.g. GitHub). See the Nature Portfolio guidelines for submitting code & software for further information.

Data

Policy information about availability of data

All manuscripts must include a data availability statement. This statement should provide the following information, where applicable:

- Accession codes, unique identifiers, or web links for publicly available datasets
- A description of any restrictions on data availability
- For clinical datasets or third party data, please ensure that the statement adheres to our policy

UniProt human FASTA database (Canonical, Version 2022-07-27, 20,420 sequences, https://www.uniprot.org/proteomes/UP000005640), BaderLab Genesets (Human_GOBP_AllPathways_no_GO_iea_December_01_2023_symbol.gmt, https://baderlab.org/GeneSets), Enrichr-KG (https://maayanlab.cloud/enrichr-kg), Reactome pathway database (https://reactome.org/), Gene Ontology (GO) database (http://geneontology.org/). The raw mass spectrometry and search result data are accessible in the MASSIVE proteomics data repository via the link: ftp://MSV000097042@massive.ucsd.edu. Data tables from all analysis is provided in the Supplemental tables 1-8.

Research involving human participants, their data, or biological material

Policy information about studies with human participants or human data. See also policy information about sex, gender (identity/presentation), and sexual orientation and race, ethnicity and racism.

Reporting on sex and gender	Sex was indeed considered as a clinical covariate in the study design. Specifically, when matching stable and vulnerable plaques, the authors ensured that sex, alongside factors such as dyslipidaemia, diabetes, BMI, symptom status, medication, and lifestyle history, was balanced between the groups (Extended Table 1).<br><br>Furthermore, during the proteomic subcluster analyses, the study found that the media subregion exhibited a significant association with sex. In this analysis, the media subclusters (derived from the proteomic data) were significantly associated with sex (adjusted mutual information [AMI] = 0.031, P = 0.040), indicating that sex-related differences may influence the proteomic profile of the media layer of carotid plaques.<br><br>Thus, while sex was incorporated as a covariate to ensure comparability between groups, the detailed spatial proteomic analysis revealed that sex may also contribute to the molecular heterogeneity observed within the plaque media.
Reporting on race, ethnicity, or other socially relevant groupings	The manuscript does not explicitly report on race, ethnicity, or other socially relevant groupings. While sex was incorporated as a key clinical covariate (with the media subregion showing a significant association with sex), no details are provided regarding race, ethnicity, or similar social factors in the clinical metadata or analyses.
Population characteristics	The study assembled its patient cohorts with a strong emphasis on balancing key clinical covariates between stable and vulnerable plaque groups. Patients were matched on several factors, including dyslipidaemia, diabetes, body mass index (BMI), sex, symptom status (with classifications such as asymptomatic, transient ischemic attack, amaurosis fugax, or stroke), as well as medication use and lifestyle history. This rigorous matching was designed to ensure that the differences observed in proteomic profiles were reflective of underlying biological processes rather than confounding clinical factors. Notably, a majority of cases (94%) exhibited an American Heart Association (AHA) histological classification of V or higher, and 79% of the patients had severe stenosis (≥80% luminal occlusion), indicating a cohort with advanced disease. While these covariates were central to the study design and analysis, the manuscript does not report on race, ethnicity, or other socially relevant groupings. See Extended table 1.
Recruitment	Patients were recruited from individuals undergoing carotid endarterectomy (CEA) at a single institution, with recruitment based on the availability of surgical specimens and complete clinical data. From an initial pool of 524 patients, plaques and corresponding serum samples were collected and subsequently curated for quality—only those plaques with clearly identifiable fibrous cap and medial layers, as well as comprehensive clinical metadata, were included in the proteomic analyses (112 plaques for proteomics and 20 for immunofluorescence validation). Serum samples were further selected based on criteria such as sufficient volume, minimal hemolysis, and the absence of prior freeze-thaw cycles. This selection strategy, while essential for ensuring high-quality data, introduces potential selection bias, as the study cohort predominantly represents patients with advanced disease who are eligible for surgery, and may not capture the full spectrum of carotid atherosclerosis, particularly early-stage lesions. Additionally, the requirement for complete clinical data and high histological quality may further limit generalizability, potentially skewing the study population towards more severe cases.
Ethics oversight	Informed written consent was obtained from all participants, in accordance with the principles of the Declaration of Helsinki. The study protocol received approval from the local ethics committee of the Technical University of Munich (DigiMed ethics 2023-297; approval no. 2799/10), ensuring that all procedures, including the collection and use of biobank samples and clinical data, met ethical requirements and maintained patient confidentiality. Clinical data were retrieved from anonymised electronic patient records, further safeguarding the privacy of the study subjects.

Note that full information on the approval of the study protocol must also be provided in the manuscript.

Field-specific reporting

Please select the one below that is the best fit for your research. If you are not sure, read the appropriate sections before making your selection.

☒ Life sciences ☐ Behavioural & social sciences ☐ Ecological, evolutionary & environmental sciences

Life sciences study design

All studies must disclose on these points even when the disclosure is negative.

Sample size	A formal a priori sample size calculation was determined using power calculation, and the availability of high-quality surgical specimens and complete clinical data, while ensuring a balanced distribution of key covariates between stable and vulnerable plaques. From an initial pool of 524 patients undergoing carotid endarterectomy, the study ultimately included 112 plaque samples—selected for histological quality (e.g., clear delineation of the fibrous cap and medial layer) and comprehensive clinical metadata—and 505 serum samples meeting stringent quality criteria. This pragmatic approach was guided by prior work in the field, with the chosen numbers deemed sufficient to capture the molecular heterogeneity of advanced plaques and to provide robust statistical power for detecting differentially abundant proteins and building predictive models. The robustness of the findings—illustrated by significant results in differential protein analyses (after multiple testing corrections), unsupervised clustering, and machine learning models (with tissue-based panels achieving an AU-ROC of 0.86)—further supports the adequacy of the sample size for addressing the study's objectives.
Data exclusions	Data exclusions were implemented at several stages to enhance data quality and ensure robust downstream analyses. For instance, in the tissue proteomics dataset, proteins with fewer than 27 observations and samples with fewer than 1,500 quantified proteins were excluded, thereby minimizing the impact of missing data and reducing statistical artifacts. Similarly, proteins with more than 66% missing values were removed to ensure that only reliable protein measurements were included. In the serum proteomics workflow, peptides detected in fewer than 25% of samples and samples with fewer than 6,000 quantified peptides were excluded, further ensuring the robustness of quantification.
Replication	All tissue samples were processed in a single batch using a semi-automated, high-throughput protocol with standardized liquid handling to minimize batch effects, while stringent quality filtering ensured that only high-confidence protein quantifications were included. Robust data processing methods—such as random forest-based imputation for missing values and cross-validation coupled with permutation testing during machine learning analyses—further reinforced the statistical validity of the results. Importantly, the predictive protein signatures identified in tissue were independently validated by quantitative immunofluorescence in an external cohort of 20 plaques, confirming that the observed proteomic differences were reproducible. Additionally, the serum proteomics approach employed a two-cohort design (a matched plaque-serum cohort and an independent cohort) to verify the consistency of vulnerability-associated protein signatures. Mechanistic findings from in vitro experiments with vascular smooth muscle cells, including controlled treatments and replicates, further substantiated the reproducibility of the observed cellular responses.
Randomization	A custom randomisation protocol was employed to distribute the samples evenly across processing batches and minimize potential batch effects. Specifically, since the plaque samples were stratified into distinct subregions (media, necrotic core, and fibrous cap) and distributed across four plates, a custom Python script was used to track and randomise the order of samples during plate preparation. This ensured that each plate contained a balanced representation of samples from both stable and vulnerable plaques, as well as a mix of other clinical covariates (such as sex and other relevant patient characteristics). Such randomisation helps to control for technical variability that could otherwise confound the differences attributed to biological factors.
Blinding	The experimentalists were blinded to the sample vulnerability status only during laser microdissection and sample processing steps in the wet-lab.

Reporting for specific materials, systems and methods

We require information from authors about some types of materials, experimental systems and methods used in many studies. Here, indicate whether each material, system or method listed is relevant to your study. If you are not sure if a list item applies to your research, read the appropriate section before selecting a response.

Materials & experimental systems

n/a	Involved in the study	
☐	☒	Antibodies
☐	☒	Eukaryotic cell lines
☒	☐	Palaeontology and archaeology
☒	☐	Animals and other organisms
☒	☐	Clinical data
☒	☐	Dual use research of concern
☒	☐	Plants

Methods

n/a	Involved in the study	
☒	☐	ChIP-seq
☒	☐	Flow cytometry
☒	☐	MRI-based neuroimaging

Antibodies

Antibodies used	See Supplementary table 8 and the method section, and their usage details.
Validation	Only commercially available antibodies were used, and validation originates from the supplier's website. The used dilutions are available in the table in the method section. All antibodies were validated for IF usage.

Eukaryotic cell lines

Policy information about cell lines and Sex and Gender in Research

Cell line source(s)	HCtASMC, PeloBiotech (PB-3514-05a, Lot: 3003, Planegg, Germany), Male human donor
Authentication	Smooth muscle specific alpha-actin antigen expression: Positive
Mycoplasma contamination	Oligonucleotide-directed amplifications: Negative/Not detected
Commonly misidentified lines (See ICLAC register)	*Name any commonly misidentified cell lines used in the study and provide a rationale for their use.*

Plants

Seed stocks	*Report on the source of all seed stocks or other plant material used. If applicable, state the seed stock centre and catalogue number. If plant specimens were collected from the field, describe the collection location, date and sampling procedures.*
Novel plant genotypes	*Describe the methods by which all novel plant genotypes were produced. This includes those generated by transgenic approaches, gene editing, chemical/radiation-based mutagenesis and hybridization. For transgenic lines, describe the transformation method, the number of independent lines analyzed and the generation upon which experiments were performed. For gene-edited lines, describe the editor used, the endogenous sequence targeted for editing, the targeting guide RNA sequence (if applicable) and how the editor was applied.*
Authentication	*Describe any authentication procedures for each seed stock used or novel genotype generated. Describe any experiments used to assess the effect of a mutation and, where applicable, how potential secondary effects (e.g. second site T-DNA insertions, mosiacism, off-target gene editing) were examined.*
