# Supplementary material for: Proteomics reveals spatial and molecular heterogeneities in advanced atherosclerotic carotid artery plaques
Source: Nat Cardiovasc Res. 2026 Jun 22;5(7):605–23. doi: 10.1038/s44161-026-00827-1 (PMC13368601; doi:10.1038/s44161-026-00827-1)
Supplement: Supplementary file 9 — Immunofluorescence images of TnC and TkC plaques obtained using protein panel. [file 44161_2026_827_MOESM9_ESM.pdf]

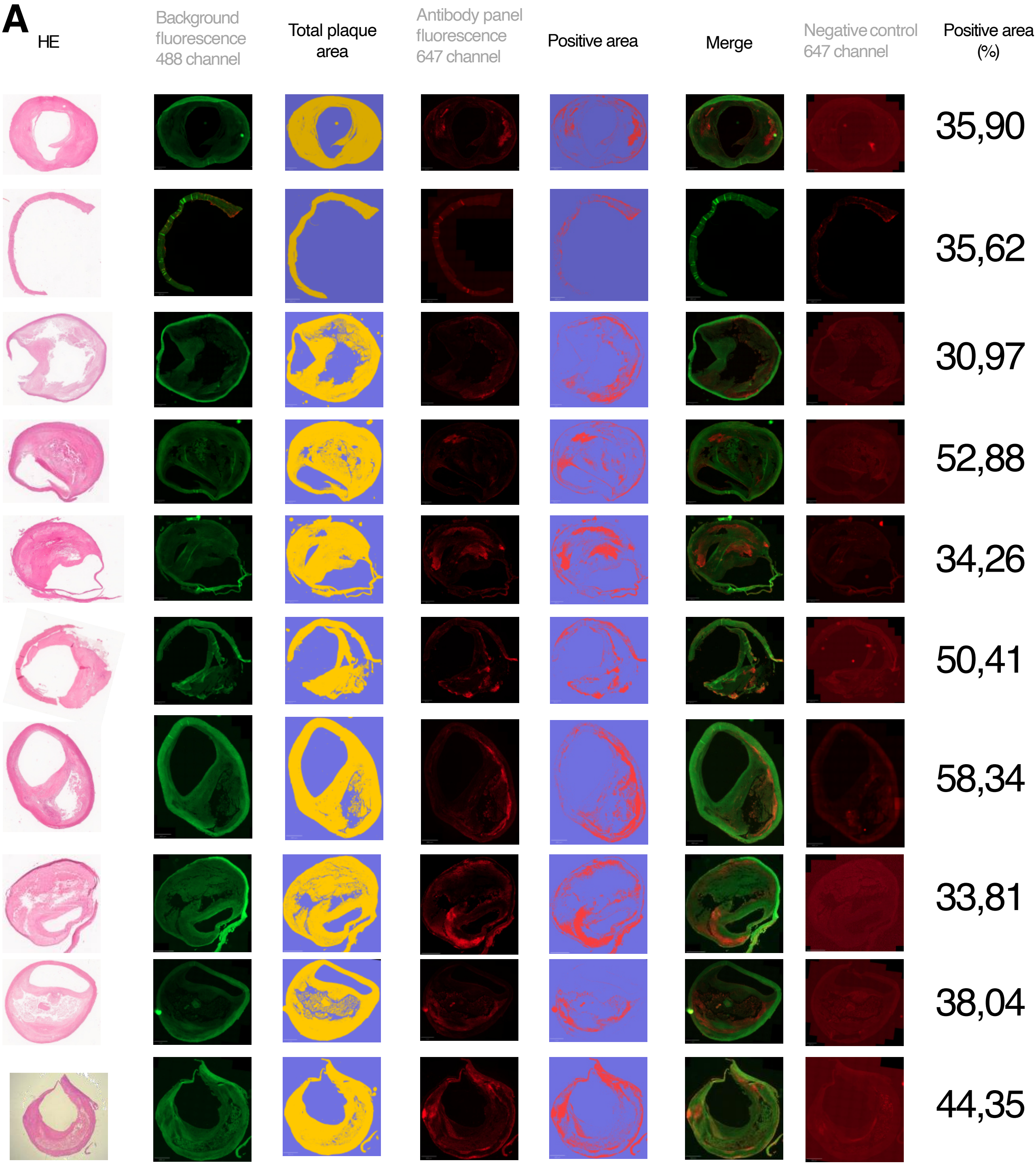

Source data for Figure 7F. Representative images showing multiplexed immunofluorescence analysis of n=20 independent plaque samples. TkC plaques are shown in **A**, and TnC plaques are shown in **B**. For each sample, sequential columns show: H&E staining for tissue morphology, tissue autofluorescence (488 channel, green), computed total plaque area mask (yellow overlay on blue background), protein panel immunofluorescence (647 channel, red), segmented positive staining regions (red overlay on blue background), merged channels, and antibody negative control (647 channel). Positive area (%) indicates the proportion of total plaque area showing protein panel expression. Images demonstrate consistent protein panel detection across samples with minimal background signal in negative controls. controls.

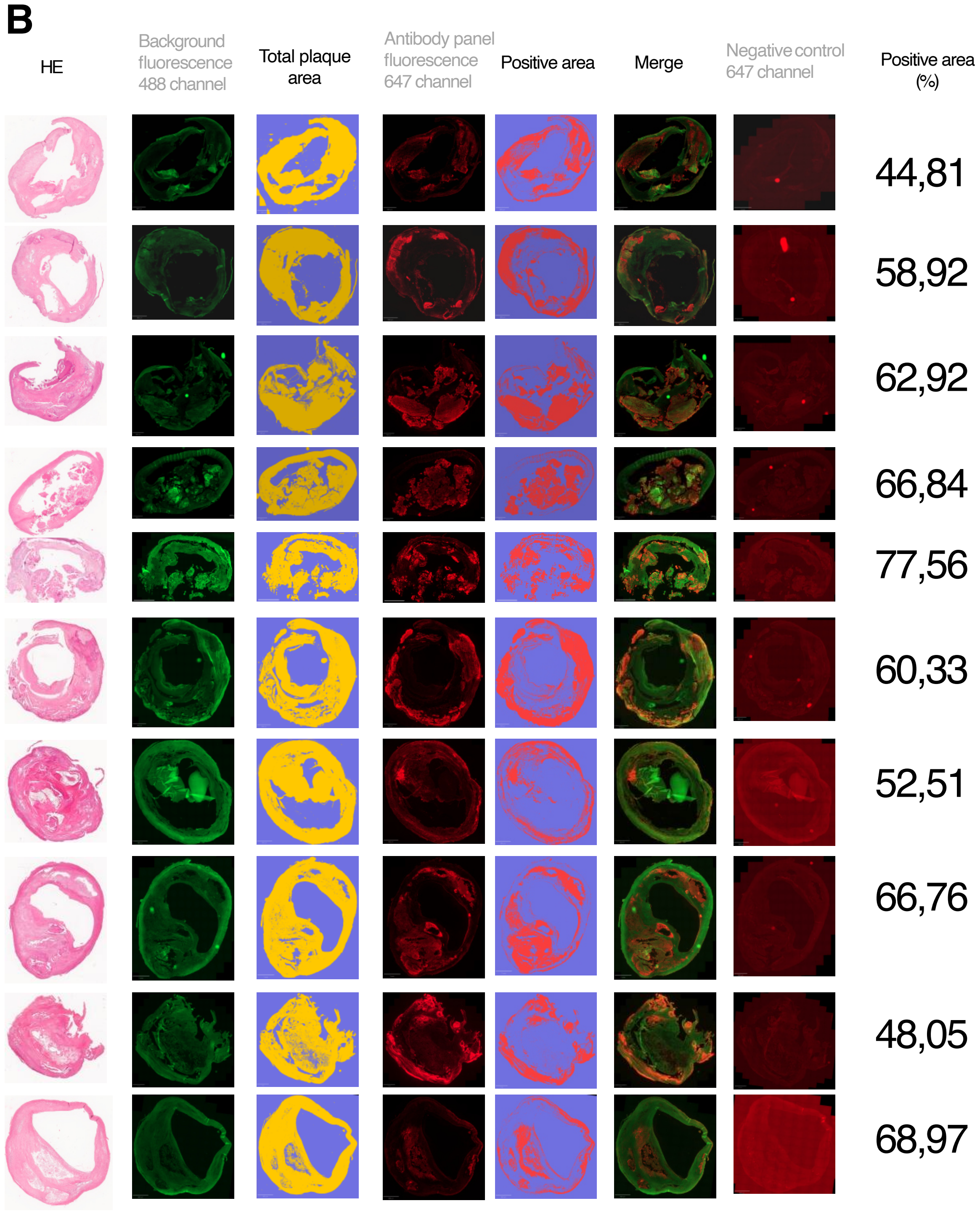
B
HE
Background fluorescence 488 channel
Total plaque area
Antibody panel fluorescence 647 channel
Positive area
Merge
Negative control 647 channel
Positive area (%)
44,81
58,92
62,92
66,84
77,56
60,33
52,51
66,76
48,05
68,97
